# Bacterial disease in seaweed aquaculture: pathogen diversity and future solutions

**DOI:** 10.1007/s10811-026-03835-7

**Published:** 2026-03-31

**Authors:** Shauna Corr, Chris Lowe, Michiel Vos

**Affiliations:** 1 European Centre for Environment and Human Health, Environment and Sustainability Institute, University of Exeter Medical School, Penryn, TR10 9FE UK; 2 Marine Ecology and Biodiversity, Plymouth Marine Laboratory, Plymouth, PL1 3DH UK; 3 The Cornish Seaweed Company Ltd, Rosuick Farm, St Martin, Helston, TR12 6DZ UK

**Keywords:** Infectious algal diseases, Bacterial pathogens, Macroalgae, Beneficial bacteria, Future solutions, Aquaculture, Marine

## Abstract

In recent years, there has been a rapid expansion in seaweed aquaculture due to the increased demand for sustainable food, pharmaceuticals, and other industrial needs. This growth has been accompanied by a rise in disease prevalence, jeopardising the economic viability of seaweed farming and the livelihoods of millions reliant on the sector. Here, through focusing on the complex interactions between seaweed hosts and their microbial communities, we review current knowledge on bacterial seaweed pathogens. We discuss the challenges associated with pathogen identification, including the constraints of traditional approaches and the complexities arising from resident commensal bacteria. We then assess the use of probiotics, selective breeding, Integrated Multi-Trophic Aquaculture (IMTA), site selection, and the priming of early life stages as emerging disease management strategies, while highlighting the need for omics-based studies to identify disease biomarkers and commercially relevant disease-resistant strains. We conclude by underlining the importance of early detection systems and improved knowledge dissemination within the sector to enable a more proactive and sustainable approach to disease management.

## Introduction

Seaweeds (macroalgae) are a diverse group of photosynthetic organisms that inhabit coastal regions worldwide (Lüning 1991). Although cultivated historically, seaweed aquaculture has intensified since the 1950 s and has been actively pursued for use in the food, nutraceutical, pharmaceutical, and cosmeceutical industries (FAO 2018; Lomartire et al. 2021). Recently, the term “phyconomy” has been proposed to encompass this large-scale, economically beneficial rise in seaweed aquaculture (Hurtado et al. 2019). The sector currently accounts for 52% of mariculture (Chopin and Tacon 2021; FAO 2024), is valued at US$14–15 billion (World Bank 2023), and makes use of over 240 commercially cultivated species, only ten of which are extensively farmed (Hermans 2021; FAO 2024).

Aside from industrial uses, cultivated seaweeds also provide ecological benefits, such as serving as nurseries and breeding grounds for crustaceans and fish (Hasselström et al. 2018), reducing eutrophication (Fei 2004), and aiding in the bioremediation of heavy metals (Davis et al. 2003) and organic contaminants (Hardegen et al. 2023). Seaweed aquaculture is also hypothesised to offer other benefits such as biofuel production, climate mitigation through assimilating CO_2_ and carbonate dissolved in seawater during growth, as biofertilisers for soil enrichment providing organic matter and nutrients, and decreasing cattle methane emissions when incorporated into livestock feeds and supplements (Duarte et al. 2017; Roque et al. 2021). However, these uses all require full life-cycle and mass balance accounting to effectively establish any genuine greenhouse-gas reductions due to emissions generated in the production process (Hurd et al. 2022; Troell et al. 2022; Pessarrodona et al. 2024).

Since the beginning of the century, expansions in commercial seaweed production have led to elevated levels of diseases and pests, with major production nations encountering substantial disease outbreaks in both wild and cultivated populations (FAO 2018). In some cases, these outbreaks have resulted in the loss of up to 50% of seaweed biomass (Ward et al. 2020; Spagnuolo and Genovese 2024; Zhang et al. 2024) and 30–50% of total farming costs (Kim et al. 2014). As millions from coastal communities rely on income from this sector (FAO 2020), disease outbreaks have significant social and financial repercussions. For instance, in the Philippines alone, outbreaks of ice-ice disease (IID), which affects tropical seaweeds such as *Kappaphycus* and *Eucheuma,* caused an estimated annual loss of US$100 million (Ward et al. 2020, 2022). Climate change has also been shown to increase the range, prevalence, and severity of bacterial diseases, while simultaneously compromising host metabolism and immune responses (Harvell 1999; 2002; 2009; Tracy et al. 2019). To develop future-proof disease management strategies, it is therefore crucial to have a comprehensive understanding of the diversity of current bacterial diseases, the factors determining disease susceptibility of hosts and possible preventative measures.

Previous reviews have focused on the varied effects of invertebrate, oocyte, virus, protist, and epiphyte diseases in seaweeds (Largo 2002; Egan et al. 2014; Egan and Gardiner 2016; Ward et al. 2020, 2022; Behera et al. 2022; Spagnuolo and Genovese 2024). Here we assess diseases caused by bacterial pathogens as they form an emerging threat for both cultivated and wild populations and pose unique management challenges due to their genetic plasticity and complex relationship with the host. We begin by giving a broad overview of the functioning of healthy seaweeds and the known diversity of seaweed bacterial pathogens. We place particular emphasis on the role commensal bacteria play in disease progression, highlighting how environmental changes can cause resident bacterial species to shift from beneficial to pathogenic roles, shaping the onset and severity of disease. We next review current and emerging solutions in seaweed disease management, focusing on the protective role of the microbiome and up-and-coming technologies.

### The seaweed holobiont

Through producing organic compounds which act as nutrients and chemo-attractants, seaweeds create metabolic niches (de Oliveira et al. 2012), which are utilised by a variety of microorganisms, some of which form symbiotic relationships with the host, establishing as an integrated, functional unit, otherwise known as the holobiont (Egan et al. 2013; Simon et al. 2019; Saha et al. 2024; Qui-Minet et al. 2025). The primary colonisers of this holobiont are usually bacteria, owing to their rapid growth rates and capacity to metabolise algal secretions (Wahl 1989; Lachnit et al. 2009). Once established, bacterial symbionts play essential roles in nutrient acquisition (Croft et al. 2005; Wahl et al. 2012), the induction of growth and morphogenetic factors (Provasoli and Pintner 1980; Matsuo et al. 2003, 2005), and in deterring fouling organisms and pathogen colonisation via the production of antifouling (Armstrong et al. 2001; Dobretsov and Qian 2002) and antimicrobial compounds (Zheng et al. 2005). The production of these secondary metabolites can also increase the host’s resilience to environmental stressors by mitigating oxidative damage and host immune responses (Dittami et al. 2016; Ghaderiardakani et al. 2020) and thus further contribute to disease resistance (Li et al. 2022a). Consequently, seaweed health is often linked to the host’s ability to recruit and regulate bacteria necessary for successful functioning under adverse conditions (Kessler et al. 2018; Ghaderiardakani et al. 2020; Hmani et al. 2024).

How similar microbiomes are across different individuals, specifically, whether individuals share a ‘core microbiome’ remains under debate (Vos 2023). Core microbiomes have been identified in *Gracilaria vermiculophylla* across seasonal scales (Mudlaff et al. 2025) and in *Laminaria hyperborea*, *Saccharina latissima* and *Durvillaea* spp. (King et al. 2023; Pearman et al. 2024) across spatial scales, however the proportion of core taxa appears to be low. This may, at least in part, reflect functional redundancy, whereby different taxa perform similar metabolic roles (Vos 2023).

**Fig. 1:**
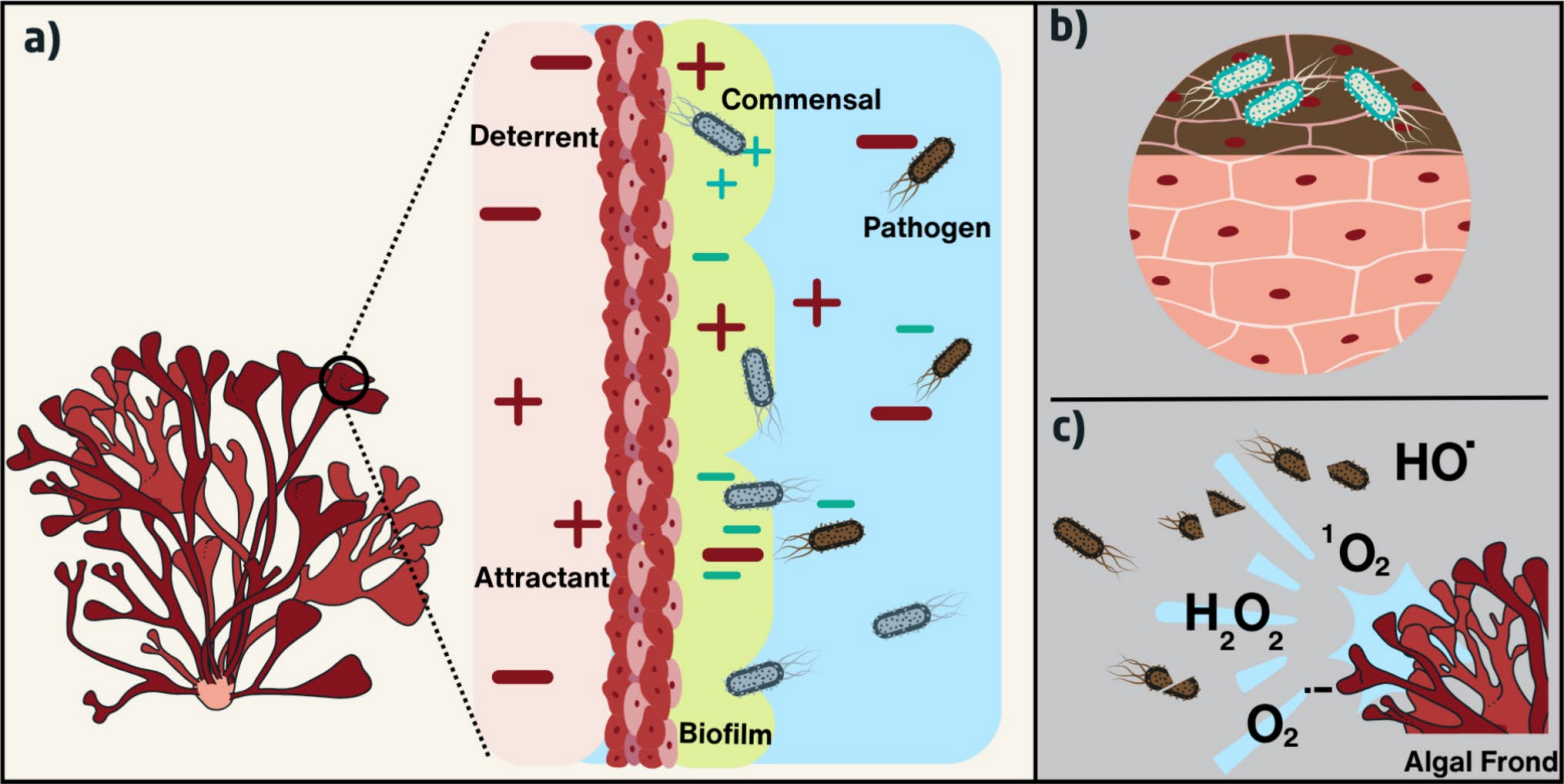
Innate Defence Mechanisms of the Seaweed Holobiont. Seaweeds have evolved a variety of innate constitutive and inducible defences against bacterial infection. **a **Constitutive holobiont defences include the production of chemical compounds by the seaweed host and/or its associated microbial symbionts, which deter potential pathogens or attract other microbiome members. **b **The hypersensitive response, an inducible form of programmed cell death, limits pathogen spread adjacent to the infection site. **c **Oxidative bursts, another inducible innate response involving the rapid release of reactive oxygen species (ROS) that can inhibit pathogens or activate downstream defence pathways

To exert control over their microbiome and combat disease, seaweeds possess a range of innate immune defences (Fig. 1) (Weinberger 2007). For example, seaweeds such as *Laurencia dendroidea* and *Delisea pulchra* have been shown to express a broad spectrum of gene transcripts associated with the biosynthesis of defensive compounds such as terpenoids and halogenated furanones, indicating a constitutive, non-specific response to their microbiome members (Manefield et al. 2011; de Oliveira et al. 2012; Lang et al. 2024). Seaweeds therefore seem to follow the “ecosystem on a leash” paradigm in which their microbiome is strongly influenced by host control (Wilde et al. 2024). Through the production of antimicrobial compounds, seaweeds can regulate their microbiome by impeding bacterial settlement (Maximilien et al. 1998), growth (Maximilien et al. 1998; Dworjanyn et al. 2006), quorum sensing (González and Keshavan 2006; Harder et al. 2012) and weakening pathogen virulence (Da Gama et al. 2014; Egan et al. 2014). Consequently, seaweeds can be highly selective toward bacteria, a selectivity that can be further influenced by the antimicrobial action of the bacteria themselves (Wiese et al. 2009).

Seaweeds possess both constitutive barriers and inducible innate responses to infection (Weinberger 2007). Unlike the cell-based immune systems seen in other organisms, seaweeds use microbe-associated molecular patterns (MAMPs) and pathogen-induced molecular patterns (PIMPs) to differentiate beneficial and pathogenic microbes (Zipfel and Felix 2005; Weinberger 2007). Upon detection of these molecular signals, seaweeds initiate signal transduction cascades that increase the expression of defence-related proteins, energy conversion, and the biosynthesis of reactive oxygen species (ROS) (Küpper et al. 2001, 2009; Weinberger 2007; de Oliveira et al. 2017). These pathways ultimately activate key innate defence mechanisms, such as oxidative bursts, cell shedding, and hypersensitive responses at infection sites (Weinberger 2007; Li et al. 2024).

Reactive oxygen species (ROS), released during oxidative bursts, are frequently used by seaweeds to counter bacterial infections through exerting cytotoxic effects on invading pathogens or by triggering further defensive responses (Weinberger 2007). Similarly, by using hypersensitive responses, seaweeds can limit the spread of pathogens by destroying cells adjacent to infection sites (Weinberger and Friedlander 2008). Although this method is effective, if used excessively, the host’s susceptibility to disease can increase due to either pathogen resistance or physiological damage to the host (Egan et al. 2014). This is often exacerbated in environments such as high-density aquaculture, where seaweeds experience increased pathogen exposures and repeated immune activations. These conditions increase the cumulative cost of host defence, compromising host health while increasing the risk of disease.

### The diversity of bacterial pathogens of seaweeds

Although many studies have reported disease symptoms or syndromes in seaweeds (Craigie and Correa 1996; Gachon et al. 2010), relatively few have conclusively identified specific causative pathogens, mechanisms of action, or possible environmental risk factors. Establishing causality typically relies on frameworks such as Koch’s postulates, which require the pathogen to be present in all instances of disease, isolatable in pure culture, able to induce identical disease symptoms in healthy specimens and be re-isolated from these infected hosts (Koch 1891). In addition, misattribution of disease symptoms is common, especially when disease expression varies markedly between hosts (Largo 2002). For instance, a single pathogen might induce rapid tissue necrosis in one host species under certain environmental conditions, whilst causing only mild discolouration or no visible symptoms in other species or environments (Largo et al. 1995a, 1995b; Gachon et al. 2010). Due to the diverse and largely unculturable nature of microbial communities, possible asymptomatic or polymicrobial nature of infection, and experimental challenges, these postulates are usually not applied to seaweeds.

Distinguishing specialised pathogens from more opportunistic species remains a major challenge in the study of seaweed diseases (Egan et al. 2013). While specialised (‘primary’) pathogens can initiate disease in healthy hosts, opportunistic pathogens are usually commensal, causing disease only when the hosts are compromised. Opportunistic pathogens can further be distinguished from secondary colonisers which do not initiate disease but rather colonise decaying tissue (Egan et al. 2013). A well-documented example involves bacteria such as *Flavobacterium*, *Pseudoalteromonas*, and *Vibrio*, which were once thought to cause green-spot disease in *Pyropia* spp. (Sunairi et al. 1995) but are in fact secondary colonisers following a viral infection by PyroV1 (Kim et al. 2016). To account for the difficulty in applying a consistent pathogen definition, we here follow the pragmatic approach taken by Bartlett et al. (2022) designating seaweed pathogens when they are isolated from a symptomatic infection and considered to be the causative agent by the reporting researchers.

The majority of bacterial pathogens implicated in seaweed disease belong to the *Alteromonas, Pseudomonas, Pseudoalteromonas* and *Vibrio* genera within the Pseudomonadota (formerly gamma-Proteobacteria) phylum (Table 1). Many of these taxa are already recognised as pathogens in other aquaculture systems, such as the Pacific white-leg shrimp (Alfiansah et al. 2020; Arunkumar et al. 2020). It is clear that commercial interest has been a main driver of pathogen identification. Consequently, most studies focus on members of the Rhodophyta inhabiting tropical regions, with extensive investigations into genera such as *Eucheuma* and *Kappaphycus* due to the widespread prevalence and economic impact of ice-ice disease (Table 1; Largo 2002). In contrast, few bacterial pathogens have been identified in temperate climes. Of note is the apparent absence of confirmed bacterial pathogens affecting Chlorophyta hosts, which likely reflects the under-representation of this phylum among cultivated seaweeds (Nayar and Bott 2014).

**Table 1:** An overview of the diversity of suspected bacterial pathogens of seaweeds, including their recorded hosts, confounding variables and possible mechanisms of action. Suspected pathogens were only included if bacteria were identified to at least genus level and were able to establish the same disease symptoms upon reintroduction to healthy specimens; the concentration of inoculum is also stated. This table excludes descriptive studies or those investigating community-level changes within diseased specimens without interrogation of Koch’s postulates. Host species belonging to wild populations are denoted with an asterisk (*)

Disease	Identified Pathogen	Identified Seaweed Hosts	Location	Symptoms	Risk Factors	Experimental Inoculation Conc	Comments	References
Suminori Disease	*Actinomycetota: Arthrobacter tumbae DY1219-4Y* *Bacteroidota: Gaetbulibacter saemankumensis H-14LY, H-15LY & H-16LY*	*Pyropia yezoensis*	Japan	Black lustreless colouration and plasmoptysis of thalli cells, reduces economic value	> Low temperatures > Low salinity	1.6 −8.3 × 10^7^ CFU mL^−1^	Mitigation: Exposure of nets to air and acid washing at pH2. Bacteriophage “U2” can repress *Gaetbulibacter* sp. H-14LY	Kusuda et al. (1992); Mine et al. (2009); Mine et al. (2010)
Anaaki Disease (Alternative Names: *Pin-Hole, Green Spot & Green Rot Disease)*	*Bacteroidota: Flavobacterium* sp*. LAD-1*	*Pyropia yezoensis*	Japan, Korea	Pin holes appear at the centre of the thallus, gradually widening into lesions with wide green borders, causing fragmentation of the thallus. Slimy rots, holes in blades, cession of growth may also appear. Degradation of the whole plant can occur as quickly as 1–2 days	> Low temperatures > Surface wounds > Ammonia availability	1 × l0^7^ CFU mL^−1^	Mitigation: Acid dipping infected thalli and exposure of nets to air and acid washing Mechanism: Bacteria with hydrolytic activity toward porphyrin, β‐D‐galactosidase, carboxymethyl cellulase, xylanase, protease and agarase activity	Fujita (1990); Sunairi et al. (1995); Kim et al. (2014)
	*Pseudomonadota: Pseudomonas* sp*. JY38*	*Porphyra dentata*	Korea			1 × 10^8^ cells mL⁻^1^		Park et al. (2001)
	Unspecified bacterium “*DOR*” isolate	*Chondrus crispus*	Canada			Cultures were "quite turbid"		Craigie and Correa (1996)
Rotten Thallus Syndrome (Alternative Names: *Thallus Whitening & Bleaching Stipe Disease)*	*Pseudomonadota: Pseudoalteromonas gracilis B9*	*Gracilaria gracilis*	Philippines, South Africa	White to pink discolouration and gradual disintegration of thallus. Erosion of the pericarp, revealing the cortical and medullary cells	> Reduced water flow > High temperatures	1 × 10^9^ CFU mL-1	Mitigation: Bacteria sensitive to polymyxin B, nalidixic acid, nitrofurazone and oxytetracycline Mechanism: Plasmid pDA1 responsible for agarolytic activity	Lavilla-Pitogo (1992); Schroeder et al. (2003)
Tip Bleaching/Whitening Stripe Disease	*Pseudomonadota:* *Vibrio parahaemolyticus, Thalassospira* spp.	*Gracilariopsis lemaneiformis*	China	Thallus whitening and apical necrosis, disintegration of thallus	> High culture density > Low light intensity > Low salinity	40,000 cells mL⁻^1^	Mitigation: 1-octen-3-one, E-2-nonenal, and benzaldehyde thought to be characteristic metabolites of infection and could be used as biomarkers for the disease	Sun et al. (2012)
White Tip Disease	Unspecified Bacterium Strain *OR-11* *Bacteroidota:* *Flavobacterium-Cytophaga* complex *Actinomycetota:* *Corynebacterium-Arthrobacter* complex	*Gracilaria conferta*	Israel	White necrotic tips followed by disintegration of thallus	> High temperature > High light intensity > High culture density > Poor aeration	2.11 × 10^7^ cells mL⁻^1^; 1:8554 ratio of cells	Mitigation: Disease could be prevented with Rifampicin, Vancomycin and Cefotaxim. Other epibacteria could also prevent bleaching	Weinberger et al. (1994), Weinberger (1997); (1999)
Bleaching Disease	*Pseudomonadota:* *Nautella* sp*. R11, Phaeobacter* sp*. LSS9,* *Alteromonas* sp*. BL110,* *Agarivorans* sp*. BL7* *Bacteroidota:* *Aquimarina* sp*. AD1 & BL5*	Delisea pulchra*	Australia	Line of bleaching from infection site. Loss of pigment causes lower photosynthetic, reproductive abilities and susceptibility to grazing, tissue necrosis and death	> High UV > High temperatures	1 × 10^6^–10^7^ CFU mL^−1^ 1 × 10^6^ cells mL⁻^1^	Mechanism: *Nautella* sp*. R11* penetrates epidermis and cortical cells causing necrosis. Pathogenicity is helped by possessing adhesion mechanisms, algal metabolite transport systems, enzymes to resist oxidative stress, cytolysins, LuxR gene varR and RaiR regulator for coordinating virulence factors. Other bacteria have strong agarase activity, phycobiliprotein can also decrease on infection	Case et al. (2011); Fernandes et al. (2011); Gardiner et al. (2015a); (2015b); Kumar et al. (2016); Hudson et al. (2018)
	*Pseudomonadota: Agarivorans albus* *Bacteroidota:* *Aquimarina latercula* *Actinomycetota:* *Brachybacterium* sp*.*	*Gracilaria lemaneiformis*	China			1 × 10^7^ CFU mL^−1^		Liu et al. (2019)
Scraping White Disease ((Alternative Names: *Bleaching Disease & White Strip Disease)*	*Pseudomonadota:* *Phaeobacter italicus* JN-W1	*Porphyra *sensu lato* conchocelis*	China	White spots, strips or patches on shells and lesions and white patched on thalli		1 × 10^7^ CFU mL^−1^	Mitigation: Pathogen can be inhibited under low temperature and pH Mechanism: Infection is contact dependent	Liu et al. (2025)
Coralline Lethal Orange Disease (CLOD)	*Bacillota:* *Planococcus citreus, Planococcus* sp., *Planococcus mcmeekinii, Bacillus pumilus* *Pseudomonadota:* *Pseudomons oleovorans*	Crustose algae*, Porolithon* spp.*	Cook islands, Fiji South Pacific reefs, Hawaii)	Occurs as a front of bright orange dots that move across the host, leaving behind a bleached carbonate skeleton	> High temperatures	Not provided	Mitigation: High wave energy habitats may inhibit attachment of CLOD propagules	Littler and Littler (1994), (1995); Cervino et al. (2005); Aeby (2007)
Yellow Spot Disease	*Pseudomonadota:* *Vibrio mediterranei* 117-T6	*Pyropia haitanensis conchocelis*	China	Yellow spots appear on infected shell-borne conchocelis sporeling stage which spread into lesions	> High temperatures > High light intensity > Low pH	6 × 10^7^ CFU mL^−1^	Mechanism: Bacterium secretes algicidal substances	Yang et al. (2020); Xu et al. (2020)
Macular Disease/Pseudomonas-like Bacteriosis	*Pseudomonadota:* *Pseudomonas-*like bacteria	*Porphyra* spp*.*	Japan	Small red/brown spots on conchoecelis of sporelings before turning yellow/white. Turns *Porphyra* gametophytes green	> High temperatures	1 × 10^7^ CFU mL^−1^	Mitigation: Discarding infected shells, chlorine treatments or increasing water flow, some sensitivity to nifurstyrenate and erythromycin	Yamanoi and Takami (2008)
Gall Formation	*Pseudomonadota: Roseobacter* group	Prionitis lanceolata; Prionitis filiformis; Prionitis decipiens*	North & South America	Gall like tumours consisting of hypertrophied algal cells		Not provided		Ashen and Goff (1996, 2000)
Ice-Ice Disease (IID)	*Pseudomonadota:* *Vibrio* sp*. P11, Vibrio* sp*. ABI-TU15, Vibrio alginolyticus, Alteromonas macleodii, Aurantimonas coralicida, Shewanella haliotis, Stenotrophomonas Maltophilia, Brucella anthropic, Catenococcus thiocycli, Pseudomonas aeruginosa* *Bacteroidota:* *Cytophaga* sp. *P25-Flavobacterium* complex *Actinomycetota:* *Arthrobacter nicrotiannae* *Bacillota:* *Bacillus subtilis*	*Kappaphycus alvarezii*	Philippines, Indonesia, India, Japan, Malaysia	Whitening and hardening or softening of tissue leading to disintegration. There can also be mucus discharge	> High light intensity > High temperatures > Low salinity > Stressed hosts > Low water movement > High phosphate concentrations > Epiphyte infestations	1 × 10^3^–10^8^ cells mL⁻^1^ 1 × 10^6^ CFU mL^−1^	Mitigation: Farm seaweeds in areas with strong water exchange, *Vibrio* ABI-TU15 growth can be inhibited by *Halomonas* species Mechanism: Hydrolytic enzymes utilised to penetrate medulla, causing epidermal degradation and destruction of pigmented plasmids	Uyenco et al. (1980); Largo et al. (1995a, (1995b), (1999); Achmad et al. (2016); Irmawati and Sudjiro (2017); Syafitri et al. (2017); Azizi et al. (2018); Zainuddin et al. (2019); Abdul Malil (2022)
	*Pseudomonadota:* *Vibrio* sp*. P11* *Bacteroidota:* *Cytophaga* sp*. P25-Flavobacterium* complex	*Eucheuma denticulatum*	Philippines			1 × 10^3^–10^5^ cells mL⁻^1^		
	*Pseudomonadota:* *Pseudomonas* spp., *Vibrio* spp., *Chromobacterium* spp*., Achromobacter* spp*.* *Bacteroidota:* *Flavobacterium* spp*.*	*Gracilaria verrucosa*	Indonesia			1 × 10^6^ CFU mL^−1^		
	*Pseudomonadota:* *Vibrio owensii*	Halymenia floresii*	Mexico			Addition of 30 μL of cultured bacteria		
Bleaching Disease	*Pseudomonadota:* *Pseudoalteromonas piscicida X-8, Alteromonas* sp*. A-1, Alteromonas espejiana*	*Saccharina japonica*	China	Bleached white tips, swollen gametophyte cells, filamentous bleaching, loss of thalli. Blades bleach and deteriorate	> High temperatures	1–3 × 10^8^ CFU mL^−1^	Bacteria can produce alginate lyase, invade the endodermis causing cell corruption and death	Wang et al. (2006); Peng and Li (2013); Zhang et al. (2022)
Green rot disease/Falling Off disease	*Pseudomonadota:* Alginic acid-decomposing bacteria *Pseudomonas* sp*.*	*Saccharina japonica*	China	Green colouration and softening of stipe, decay leads to detachment of plant	> Insufficient light	Not provided	Mitigation: Addition of erythromycin, discard decayed blades, maintain cultures at 6–8 ०C and replacing seawater	Wu (1990); Liu et al. (2002); Wang et al. (2014)
Thallus Bleaching disease	*Pseudomonadota:* *Alteromonas* sp.	*Saccharina religiosa*	Japan	Induced lesions and thallus bleaching	> Low salinity > High temperature	3 × 10^8^ cells mL⁻^1^		Vairappan et al. (2001)
Red spot disease	*Pseudomonadota:* *Pseudoalteromonas bacteriolytica* sp.	*Saccharina japonica*	Japan	Red spotted staining of culture beds and detachment of sporophyte	> High temperatures > High POM concentrations > High levels of bacteria flora	Not provided	Mitigation: Washing “Mother” seaweed with sterilized seawater and cutting off decayed parts	Ezura et al. (1988); Sawabe et al. (1998); Yumoto et al. (1989a), (1989b)
Hole-Rotten Disease	*Pseudomonadota:* *Pseudoalteromonas* sp*., Vibrio* sp*., Halomonas* sp.	*Saccharina japonica*	China	Green rotting on sporophytic edges and holes on blade, which spread until detachment from ropes		1 × 10^7^ CFU mL^−1^		Wang et al. (2008)

Knowledge of disease outbreaks in natural populations lags behind that in aquaculture, even though the consequences can be substantial both ecologically and economically. For example, a decrease in primary production and carbonate accretion occurred due to an outbreak of coralline lethal orange disease (CLOD) affecting a vast population of reef-building coralline algae in the early 1990 s (Littler and Littler 1994, 1995). Consequently, wild populations not only have to contend with naturally occurring disease outbreaks but repercussions of the rapidly expanding aquaculture sector, such as the introduction of non-native diseases, epidemics, and a decrease in the genetic diversity of natural stocks (Spagnuolo and Genovese 2024).

Notably, fewer than half of the reported seaweed diseases here are associated with a single bacterium, highlighting the limitations of the classic “one pathogen = one disease” model (Vayssier-Taussat et al. 2014). Historically, studies have primarily concentrated on monomicrobial infections, but more recently many infections have been shown to be polymicrobial either in origin or manifestation (Tay et al. 2016). Pathogens often live in complex microbial communities where different interactions determine the progression of disease (Chow et al. 2011; Rogers 2012). This is the basis of the “pathobiome” concept, in which pathogenicity is conditional, modulated by interactions with the host, environmental factors and fellow microbiome members (Bass et al. 2019).

### Commensal bacteria: Friend or Foe?

Although infectious disease is often associated with invading pathogens, emerging evidence indicates that many diseases appear, not from external pathogens, but resident and usually benign members of the host's microbiota (Chow et al. 2011; Egan and Gardiner 2016). For instance, *Staphylococcus aureus* is usually a commensal component of the human microbiome yet can cause severe and sometimes fatal infections when host defences are compromised (Turnbaugh et al. 2009). Seaweeds are no exception, with ten of the 23 pathogens listed in Table 1 previously associated with beneficial traits in other seaweed species.

Environmental stress events often coincide with disease outbreaks in both wild and cultivated seaweeds (Campbell et al. 2011; Ward et al. 2022). For example, *Vibrio alginolyticus* strain X-2 can upregulate host genes linked to stress and immune responses and preserve epibacterial community structure in *S. japonica,* while causing disease in a diverse array of other seaweed hosts (Ma et al. 2023). Through using -omics techniques, disease states have been shown to be associated with shifts in microbial gene expression and abundance rather than the appearance of entirely new taxa (Fernandes et al. 2012; Egan et al. 2013). This suggests that pathogenicity is highly context-driven, dependent on the expression of virulence traits under certain conditions. In the plant literature, the term “eubiosis” refers to a normally functioning, homeostatic microbiome associated with a healthy individual (Paasch and He 2021), where as a disruption is often referred to as “dysbiosis” and is widely known to detrimentally impact host health (Li et al. 2022a; Ling et al. 2022). However, it must be noted that this term remains contentious due to the absence of a clear definition and its assumption of an ‘ideal’ microbial state (Hooks and O’Malley 2017; Mancuso et al. 2023).

In line with the phenotypic plasticity of many marine bacteria, the ability to shift from commensal residents to pathogens is unsurprising. Numerous taxa possess latent virulence factors which, although tightly repressed under normal conditions, can be upregulated under host stress or environmental fluctuations (Fernandes et al. 2011; Billaud et al. 2022; Bharathan et al. 2025). For seaweeds, which lack an adaptive immune system, even subtle changes in environmental conditions can disrupt the host’s surface chemistry or microbial-mediated checks which suppress virulence, permitting formerly harmless commensals to proliferate and initiate disease (Zozaya-Valdes et al. 2015).

One of the best-documented examples of dysbiosis in seaweeds is the bleaching disease of the red alga *Delisea pulchra.* In this system, changes in microbial community composition can occur prior to visible tissue damage (Fernandes et al. 2012). Several bacterial taxa usually present in the microbiome, including *Nautella italica* R11 and *Phaeobacter galleciensis* LSS9, can cause disease when host defence mechanisms are compromised, such as at elevated temperatures, often due to increases in their abundance (Case et al. 2011; Fernandes et al. 2011; Gardiner et al. 2017). Following this, the bacteria deploy virulence factors and QS-dependent regulatory mechanisms, exploiting the weakened host (Case et al. 2011; Fernandes et al. 2011; Gardiner et al. 2017). Similarly, when exposed to low light intensity and salinity, resident bacteria of *Kappaphycus alvarezii,* such as *Vibrio* sp. P11 and *Cytophaga* sp. P25, can induce ice-ice disease (Largo et al. 1995b).

The difficulty in identifying seaweed pathogens thus stems from the fact that many are not obligate pathogens but rather commensal microbiota ‘gone rogue’, shifting their behaviour to exploit weakened hosts. In the context of seaweed aquaculture, where monocultures can exacerbate microbial imbalance by permitting the rapid spread of pathogens, predicting and mitigating disease requires identifying the conditions under which commensal bacteria shift from benign association to causing opportunistic disease. Although challenging, this diagnostic complexity highlights the need for adopting a pathobiome perspective or framework that recognises that disease is a result of many complex interactions between the host, microbiome, and the wider environment (Foster et al. 2017; Bass et al. 2019). Future disease control, therefore, is most likely to make advances where it moves towards manipulating the microbial environment rather than targeting individual pathogens.

## Future directions in seaweed disease control

**Fig. 2:**
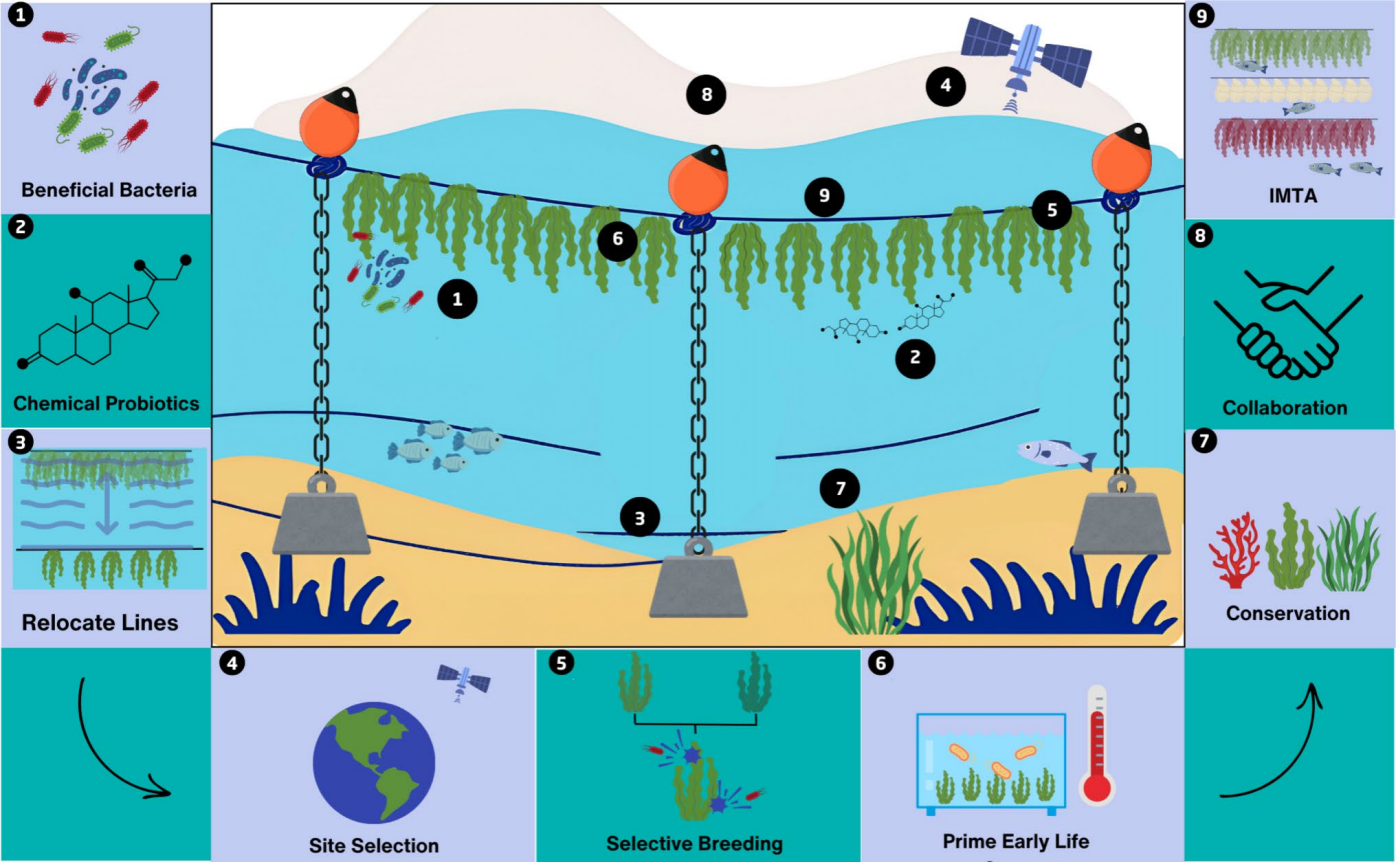
Future solutions for disease mitigation in aquaculture include (1) manipulating the microbiome to become more resilient with beneficial bacteria. (2) Using probiotics to increase host health. (3) Changes to infrastructure such as lowering cultivation lines to lower water depths in intense UV or temperatures. (4) Modelling future disease outbreaks and favourable climate conditions to optimise site selection. (5) Selectively breeding disease-resistant strains. (6) Priming early life stages to be more resilient. (7) Conserving the genetic diversity of wild populations. (8) Increased collaboration between scientists, farmers and industry to disseminate pertinent knowledge more efficiently. (9) The use of IMTA methods to reduce disease susceptibility

Due to the many health benefits of the seaweed microbiome, recent efforts to enhance host growth, environmental resilience, and disease resistance through scalable and cost-effective microbial approaches have increased in both agricultural and conservation contexts (El-Saadony et al. 2021; Peixoto et al. 2022; Voolstra et al. 2024). In the next part of this review, we explore key developments in aquaculture practises and emerging disease management research topics for this expanding sector (Fig. 2). While nanotechnology, antibiotics and acid-washing seaweed blades have shown some potential such as reducing fungal disease in rainbow trout eggs (Johari et al. 2016) or manipulating epiphytic communities to enhance seaweed biomass (Blanco et al. 2025), we do not address their application here, as the open nature of seaweed farming poses too great a risk of environmental contamination, especially when more suitable alternatives are available.

### Direct microbiome manipulation

#### Probiotics

The use of bacteria with antagonistic activity against pathogens has frequently been used to enhance the resilience of microbiomes associated with terrestrial crops (Quiza et al. 2015). For example, reducing the severity of rice blast disease by up to 70% can be achieved through enriching native bacteria such as *Streptomyces* sp. UPMRS4 and *Pseudomonas mosselii* BS011 (Awla et al. 2017; Wu et al. 2018). Such techniques could be beneficial in seaweed cultivation, where several bacteria have already been identified to increase disease resistance by enhancing host health (Li et al. 2022b; Ma et al. 2023). Although, in contrast to terrestrial crops, the application of these bacteria (also known as Seaweed Beneficial Microorganisms, SBMs (Li et al. 2023)) in seaweed aquaculture remains relatively novel.

Beneficial bacteria may prevent disease through several mechanisms: either by directly inhibiting pathogen virulence (Fan et al. 2020), monopolising resources (Gu et al. 2020), interacting with other microbiome members (Li et al. 2022a), stimulating host bioactive compound production (Wen et al. 2023), or producing antimicrobials (Granato et al. 2019). For instance, two strains of *Pseudoalteromonas piscicida,* P3 and P6, were shown to inhibit disease symptoms inflicted by *Pythium porphyrae* in *Pyropia yezoensis,* providing an effective biocontrol methodology against Red Rot Disease (Weng et al. 2024). Similarly, *Phaeobacter* sp. BS52 protects *Delisea pulchra* against the opportunistic bleaching pathogen *Aquimarina* sp. AD1 (Li et al. 2022b). Interestingly, this species was also found to diminish the detrimental effects of bleaching disease when inoculated onto non-host species (Li et al. 2022b)*.* As bleaching diseases are likely to become more common, this probiotic bacterium might be promising in enhancing host disease resistance in future ocean warming scenarios.

Two methods for probiotic application are currently being explored: either the inoculation of non-native SBMs or the reinoculation of an isolated and enriched native bacterium (Lau et al. 2022). A third possibility is the introduction of genetically modified strains, but this would require strict regulation (Ke et al. 2021). Regardless of method, further work is needed to ascertain whether introduced bacteria could be maintained in large-scale industrial settings and, if so, how they transfer between host life stages.

Environmental and biosecurity risks also require attention. Threats such as the unintended emergence of novel diseases need to be prevented, either through limiting the use of microbes to secure hatchery stages or through solely applying the microbe’s beneficial active compound, for instance, directly applying the compound thallusin rather than its bacterial producers (Dhiman et al. 2022; Ulrich et al. 2022). Similarly, prebiotics, non-digestible feed additives historically used to improve gut microbiome health in fish, can enhance nutritional uptake and resistance to disease (Dimitroglou et al. 2011; Ganguly et al. 2013). In seaweeds, such additives could be used in a similar manner, either to nourish beneficial bacteria or as potential pathogen deterrents. The enrichment of beneficial host microbiomes has already been observed under land-based cultivation and laboratory conditions thought to be mediated by intrinsic host factors such as Algal Growth and Morphogenesis-Promoting Factors (Ghaderiardakani et al. 2019; Califano et al. 2020; Nguyen et al. 2023). Seaweeds rely on metabolites with antifoulant and antimicrobial activity: for example, compounds such as halogenated furanones and fucoxanthin can mitigate surface fouling and bacterial attachment (Campbell et al. 2011; Saha et al. 2011), and 1-octen-3-one, E-2-nonenal, benzaldehyde, and oxylipins can counter bacterial disease (Sun et al. 2012). However, research into seaweed prebiotics is still in its infancy and it is still unclear whether beneficial compounds can be effectively applied or upregulated within host tissues.

#### Selective breeding

Selective breeding, which utilises controlled reproduction of individuals possessing desirable characteristics, has already been implemented for various economically important seaweeds, such as *Porphyra* and *Laminaria* species (Chaoyuan and Guangheng 1987; Dai et al. 1993). To produce characteristics such as enhanced taste and resistance to high irradiance, temperatures and tissue rot disease, hybridisations such as those of *Saccharina longissima* and *S. japonica* have also been utilised (Li et al. 2007; Robinson et al. 2013; Hwang et al. 2019).

Although widely used in terrestrial agriculture, omics techniques such as metabolomics and genomics could also be applied to seaweed cultivation (Patwary et al. 2021). These techniques could be used to mitigate disease by identifying genes, regulatory elements and metabolic pathways linked to resistant traits, including those integral to microbiome regulation, antimicrobial compound production and adaptive immune responses (DeWeese and Osborne 2021). Such genes can be incorporated into breeding programmes through marker-assisted selection or introduced via genetic engineering.

As of 2024, over 110 seaweed genomes representing 105 species are publicly available, aiding new selective breeding programmes (Wang et al. 2020; Nelson et al. 2024). These datasets enable the application of Genome-Wide Association Studies (GWASs), which explore single-nucleotide polymorphisms (SNPs) associated with desirable qualities, and Quantitative Trait Loci (QTL) mapping, which can detect the genetic loci contributing to specific phenotypes (Murray et al. 2008; Huang and Han 2014; Huang et al. 2015; DeWeese and Osborne 2021; Feng et al. 2023). Through combining these approaches, candidate genes and genomic regions linked to disease-resistant traits could be identified and incorporated into genomic predictions to develop disease-resistant seaweed strains.

To identify future commercial cultivars, transcriptomics could be used to highlight gene expression profiles connected to beneficial traits (Chin et al. 2017; Thien et al. 2020; Zhuang et al. 2024). By comparing strain-specific transcriptomes exposed to stress, beneficial traits such as elevated growth rate, disease resistance and higher carrageenan content could be identified and used to support selection and breeding decisions. For example, a substantial reservoir of differentially upregulated defensive genes, such as heat shock proteins, secondary metabolites, protective enzymes, and cellulase and protease inhibitors has already been found in *P. yezoensis* (Tang et al. 2019). Likewise, transcriptomic activation of genes connected with stress response pathways and cell-wall strengthening has been observed in a disease-resistant genotype of *S. japonica* (Zhuang et al. 2024). This approach could be further extended to investigate traits which aid the maintenance of beneficial interactions within the host’s microbiome.

While classical breeding is an effective strategy, its success is inherently restricted to traits already present in the breeding population. Genetic engineering, already used in several seaweed species, permits the introduction of traits not naturally present, such as the expression of recombinant proteins and reporter genes to produce antigens, antibodies and other therapeutical compounds (Jiang et al. 2002; Zhang et al. 2008; Badis et al. 2021; Shen et al. 2023; Trujillo et al. 2024). Farms growing genetically engineered disease-resistant strains may be operational soon, depending on their adherence to GMO and biosecurity policies. However, even though research is rapidly progressing in this area, many nations lack clear regulations governing genetically modified or selectively bred seaweed strains (Goecke et al. 2020).

#### Priming early life stages

Priming early life stages to sublethal stresses activates specific stress-responsive genes and can be used to increase resilience within cultivars (Jueterbock et al. 2021). For example, in *A. esculenta* sporophytes, exposure in early cultivation stages to high light intensity reduces thermal stress by enhancing photoprotective mechanisms (Martins et al. 2022). In other brown macroalgae, similar priming effects have been noted where thermal or oxidative stress causes persistent changes in antioxidant capacity or defence-related gene expression (Heinrich et al. 2012).Therefore, similar to abiotic stressors, exposing seaweeds to microbial and chemical cues could prime the host against subsequent pathogenic challenges.

Instead of applying direct stressors, waterborne chemical cues released by certain herbivores may stimulate the production of defensive compounds and bolster seaweeds against disease outbreaks (Toth and Pavia 2000). As seaweeds are known to utilise a broad spectrum of chemical cues to attract beneficial bacteria and suppress disadvantageous strains (Saha and Weinberger 2019), such metabolites could also be explored for potential use in recruiting beneficial bacteria. While early-life priming may offer a novel approach to mitigating disease in future commercial cultivars, there still is a lack of evidence that this strategy increases disease resistance in macroalgae. Further experimental work is needed to assess if this approach could indeed increase the defensive capacity of seaweeds against pathogenic infection.

### Practices that promote microbial eubiosis

#### Site selection and farming practises

To reduce disease outbreaks, a combination of technological advancements and improved aquaculture practises, such as suitable site selection, are needed. For example, during periods of heightened light intensity and temperature, farms could relocate lines to deeper waters, decreasing the chance of thermally induced diseases (Makame et al. 2021). Similarly, improving water flow by reducing overcrowding could aid the dispersal of beneficial microbes while simultaneously inhibiting the accumulation of opportunistic pathogens, thereby decreasing the transmission of pathogens and promoting resilient microbial communities.

However, for sustainable, long-term cultivation, effective site selection must also incorporate broader spatial areas than individual farms. To assess locations for optimal environmental parameters for both the seaweed host and its microbiome, increasingly sophisticated climate and oceanographic modelling is now available (Veenhof et al. 2024). Bathymetric and hydrodynamic analysis of a previously failed site in Lampung Bay, Indonesia, exemplifies the need for rigorous analysis in the placement of future ventures (Yulianto et al. 2017). Similar investigation of areas of low water flow or proximity to freshwater sources may also increase the likelihood of success, reducing stress and microbial disruption caused by salinity fluctuations and increased pathogen loads (Largo 2002; van der Loos et al. 2022).

Socio-economic and practical considerations are as valuable as environmental factors when assessing cultivation sites. Studies from Zanzibar and Europe have revealed the importance of aligning farming sites with the farmer’s local preferences, access to infrastructure and the area’s traditional uses (Hedberg et al. 2018; Thomas et al. 2019). Overlooking these factors may result in the abandonment or underutilisation of sites, regardless of environmental suitability. Looking ahead, the co-location of cultivation sites with offshore wind parks could be a possible solution for sharing marine space, reducing user conflicts and benefiting from the sheltering effect of existing infrastructure (Coffey et al. 2025). The integration of socio-economic data with oceanographic modelling is therefore essential for future spatial planning of farming ventures in changing climates.

#### Co-culturing and Integrated Multi-Trophic Aquaculture (IMTA)

In coastal settings, the expansion of invertebrate and fish aquaculture can cause significant shifts to water quality due to the release of elevated nutrients (Gao et al. 2021), disrupting ecosystem functioning and contributing to the spread of disease. Approaches which co-culture several species, such as *Kappaphycus* spp. with *Eucheuma denticulatum,* have already been shown to enhance resilience and decrease the prevalence of diseases such as IID in summer months (Pang et al. 2015). A meta-analysis revealed that proximity of seaweed farms to seagrass fields reduces disease risk (Fiorenza et al. 2024). It is possible that co-cultivation of commercial seaweed crops with specific other ‘companion crop’ seaweed species could equally reduce disease incidence (e.g. Fu et al. 2015), but to our knowledge this has not been tested.

The creation of a balanced ecosystem through cultivating different trophic levels, known as integrated multi-trophic aquaculture (IMTA), represents a logical step in future disease management, as multiple species provide enhanced disease resistance and help to curtail environmentally harmful practises (Troell et al. 2009; Kim et al. 2014). Seaweeds absorb nitrates, ammonia, phosphates and heavy metals, acting as natural bio-remediators; their inclusion in other aquaculture systems can therefore mitigate nutrient pollution while maintaining economic value (Gao et al. 2021; Sugumaran et al. 2022). Beyond disease mitigation, these seaweeds can provide habitat and improve water quality, ultimately benefiting higher trophic levels by enhancing food availability and providing new breeding and nursery habitats (Corrigan et al. 2024).

#### Knowledge sharing

Disease outbreaks in intensive aquaculture systems are usually multifactorial and may result from the disregard of region-specific cultivation guidelines, limited knowledge on symptoms and pathologies, low genetic variation causing decreased resilience of populations, poorly chosen cultivation sites, and/or the introduction of novel pathogens through farming non-native species (Spagnuolo and Genovese 2024). Addressing these issues requires improved collaboration among researchers, industry stakeholders, and policymakers to promote the dissemination of knowledge and best practices. Such coordination could be addressed at scientific meetings (e.g., the International Society for Applied Phycology; the International Seaweed Symposium organised by the International Seaweed Association and Seagriculture EU and Asia–Pacific), but digital tools and citizen science programmes could also play key roles. For example, the wider adoption and maintenance of platforms used to support the early detection and management of diseases, such as the web portal “My Seaweed Looks Weird”, which enabled farmers to report unusual diseases and submit samples (Strittmatter et al. 2022), could improve disease resistance and resilience across the sector.

As reservoirs of genetic diversity, wild seaweed populations offer resilience to the emerging threats of climate change and therefore must be restored and conserved (Eger et al. 2024). The mapping of genetic variation among populations with valuable cultivation and restoration traits is already underway (Mao et al. 2020). Combined with the establishment of biobanks, these approaches may preserve genetic material and create a safeguarded reserve for future biodiversity and breeding efforts (Cottier-Cook et al. 2016; Wade et al. 2020; Brakel et al. 2021; Fouqueau et al. 2024; Hofmann et al. 2025), supporting the development of robust, climate-resilient aquaculture systems.

Finally, implementing a systematic health assessment framework, such as that proposed by Work and Aeby (2006) for coral disease, could standardise disease diagnosis between regions. Through integrating microbiological and environmental contexts and providing consistent terminology and symptom documentation, such frameworks could reduce losses in aquaculture through improving health monitoring throughout the entire seaweed life cycle while enhancing differentiation between healthy and diseased specimens.

## Conclusions

Seaweed aquaculture is rapidly expanding due to its role as a sustainable food source (both as whole seaweeds and extracts used in processed foods), its support of coastal livelihoods, and its contribution to the UN Sustainable Development Goals. However, disease outbreaks increasingly endanger the resilience of this sector. Our review highlights that maintaining seaweed health requires advancing beyond a single-pathogen view and embracing the holobiont perspective, acknowledging that seaweeds and their microbial communities function as a unified system.

A core concept in disease prevention is the dual role of commensal bacteria. Although microbes provide many beneficial services such as nutrient acquisition (Croft et al. 2005), defence (Armstrong et al. 2001), and stress tolerance (Dittami et al. 2016), under environmental stressors they can shift to pathogenic behaviour, complicating diagnosis. Therefore, maintaining microbial communities instead of controlling invading pathogens may provide a next logical step in disease management. Many emerging techniques support these views, with probiotics, selective breeding, and priming of early life stages able to enhance host resilience, while better site selection could reduce the impact of environmental stressors that cause dysbiosis. Early detection and subsequent targeted interventions may now be possible due to the increase in -omics techniques available to identify biomarkers of disease and resistant strains.

Nevertheless, serious challenges remain. The increasing pressures of climate change add to the urgency in developing dependable solutions and knowledge-sharing systems across the sector. To create long-term approaches for proactive disease prevention, seaweed aquaculture will need to depend on an integrated understanding of the holobiont including host biology, microbiome dynamics, and environmental change.

## Data Availability

No datasets were generated or analysed during the current study.

## References

[CR1] Abdul Malik SA, Saha M, Taupin L, Bedoux L, Bourgougnon N, Robledo D (2022) Identification of the quorum sensing signal of the opportunistic pathogen inducing bleaching disease in the red macroalga *Halymenia floresii* holobiont. Appl Phycol 3:109–119

[CR2] Achmad M, Alimuddin A, Widyastuti U, Sukenda, S, Suryanti E, Harris, E (2016) Molecular identification of new bacterial causative agent of ice-ice disease on seaweed *Kappaphycus alvarezii*. PeerJ Preprints 4:e2016v1

[CR3] Aeby GS (2007) First record of coralline lethal orange disease (CLOD) in the Northwestern Hawaiian Islands. Coral Reefs 26:385

[CR4] Alfiansah YR, Peters S, Harder J, Hassenrück C, Gärdes A (2020) Structure and co-occurrence patterns of bacterial communities associated with white faeces disease outbreaks in Pacific white-leg shrimp *Penaeus vannamei* aquaculture. Sci Rep 10:11980 32686764 10.1038/s41598-020-68891-6 PMC7371890

[CR5] Armstrong E, Yan L, Boyd KG, Wright PC, Grant Burgess J (2001) The symbiotic role of marine microbes on living surfaces. Hydrobiologia 461:37–40

[CR6] Arunkumar M, LewisOscar F, Thajuddin N, Pugazhendhi A, Nithya C (2020) *In vitro* and *in vivo* biofilm forming *Vibrio* spp.: a significant threat in aquaculture. Process Biochem 94:213–223

[CR7] Ashen JB, Goff LJ (1996) Molecular identification of a bacterium associated with gall formation in the marine red alga *Prionitis lanceolata*. J Phycol 32:286–297

[CR8] Ashen JB, Goff LJ (2000) Molecular and ecological evidence for species specificity and coevolution in a group of marine algal-bacterial symbioses. Appl Environ Microbiol 66:3024–3030 10877801 10.1128/aem.66.7.3024-3030.2000 PMC92106

[CR9] Awla HK, Kadir J, Othman R, Rashid TS, Hamid S, Wong M (2017) Plant growth-promoting abilities and biocontrol efficacy of *Streptomyces* sp. UPMRS4 against *Pyricularia oryzae*. Biol Control 112:55–63

[CR10] Azizi A, Mohd Hanafi N, Basiran MN, Teo CH (2018) Evaluation of disease resistance and tolerance to elevated temperature stress of the selected tissue-cultured *Kappaphycus alvarezii* Doty 1985 under optimized laboratory conditions. 3 Biotech 8:321 30034985 10.1007/s13205-018-1354-4 PMC6049812

[CR11] Badis Y, Scornet D, Harada M, Caillard C, Godfroy O, Raphalen M, Gachon CMM, Coelho SM, Motomura T, Nagasato C, Cock JM (2021) Targeted CRISPR-Cas9-based gene knockouts in the model brown alga *Ectocarpus*. New Phytol 231:2077–2091 34076889 10.1111/nph.17525

[CR12] Bartlett A, Padfield D, Lear L, Bendall R, Vos M (2022) A comprehensive list of bacterial pathogens infecting humans. Microbiology 168:12 10.1099/mic.0.001269 36748702

[CR13] Bass D, Stentiford GD, Wang H-C, Koskella B, Tyler CR (2019) The pathobiome in animal and plant diseases. Trends Ecol Evol 34:996–1008 31522755 10.1016/j.tree.2019.07.012 PMC7479508

[CR14] Behera DP, Ingle KN, Mathew DE, Dhimmar A, Sahastrabudh H, Sahu SK, Krishnan GG, Shinde P, Meenakshisundaram G, Mantri VA (2022) Epiphytism, diseases and grazing in seaweed aquaculture: a comprehensive review. Rev Aquac 14:1345–1370

[CR15] Bharathan A, Arafath Y, Fathima A, Hassan S, Singh P, Kiran GS, Selvin J (2025) Implication of environmental factors on the pathogenicity of *Vibrio vulnificus*: insights into gene activation and disease outbreak. Microb Pathog 204:107591 40246153 10.1016/j.micpath.2025.107591

[CR16] Billaud M, Seneca F, Tambutté E, Czerucka D (2022) An increase of seawater temperature upregulates the expression of *Vibrio parahaemolyticus* virulence factors implicated in adhesion and biofilm formation. Front Microbiol 13:840628 35350627 10.3389/fmicb.2022.840628 PMC8957992

[CR17] Blanco S, Campbell AH, Cummins SF, Heyne DA, Paul NA (2025) Manipulation of the surface-associated bacteria on *Asparagopsis taxiformis* and its effect on seaweed growth and halogenated natural products. J Appl Phycol 37:2679–2689

[CR18] Brakel J, Sibonga RC, Dumilag RV, Montalescot V, Campbell I, Cottier-Cook EJ, Ward G, Le Masson V, Liu T, Msuya FE, Brodie J, Lim PE, Gachon CMM (2021) Exploring, harnessing and conserving marine genetic resources towards a sustainable seaweed aquaculture. Plants People Planet 3:337–349

[CR19] Califano G, Kwantes M, Abreu MH, Costa R, Wichard T (2020) Cultivating the macroalgal holobiont: effects of integrated multi-trophic aquaculture on the microbiome of *Ulva rigida* (Chlorophyta). Front Mar Sci 7:52

[CR20] Campbell AH, Harder T, Nielsen S, Kjelleberg S, Steinberg PD (2011) Climate change and disease: bleaching of a chemically defended seaweed. Glob Change Biol 17:2958–2970

[CR21] Case RJ, Longford SR, Campbell AH, Low A, Tujula N, Steinberg PD, Kjelleberg S (2011) Temperature induced bacterial virulence and bleaching disease in a chemically defended marine macroalga. Environ Microbiol 13:529–537 20946533 10.1111/j.1462-2920.2010.02356.x

[CR22] Cervino JM, Littler MM, Littler DS, Polson SW, Goreau T, Brooks B, Smith G (2005) Identification of microbes associated with coralline lethal algal disease and its relationship to glacial ice melt (global warming). Phytopathology 95:120–121

[CR23] Chaoyuan W, Guangheng L (1987) Progress in the genetics and breeding of economic seaweeds in China. Hydrobiologia 151:57–61

[CR24] Chin GJWL, Mohamad MZ, Maili S, Yong WTL, Rodrigues KF (2017) ISSR-PCR fingerprinting of *Kappaphycus* and *Eucheuma* (Rhodophyta, Gigartinales) seaweed varieties from Sabah, Malaysia. Trans Sci Technol 4:420–425

[CR25] Chopin T, Tacon AGJ (2021) Importance of seaweeds and extractive species in global aquaculture production. Rev Fish Sci Aquac 29:139–148

[CR26] Chow J, Tang H, Mazmanian SK (2011) Pathobionts of the gastrointestinal microbiota and inflammatory disease. Curr Opin Immunol 23:473–480 21856139 10.1016/j.coi.2011.07.010 PMC3426444

[CR27] Coffey B, Borgerson C, Lal P, Feehan CJ (2025) Co-location of seaweed farming with offshore wind energy: a quick scoping review. Front Mar Sci 11:1471204

[CR28] Corrigan S, Smale D, Tyler C, Brown A (2024) Quantification of finfish assemblages associated with mussel and seaweed farms in southwest UK provides evidence of potential benefits to fisheries. Aquacult Environ Interact 16:145–162

[CR29] Cottier-Cook EJ, Nagabhatla N, Badis Y, Campbell ML, Chopin T, Dai W, Fang J, He P, Hewitt CL, Kim GH, Huo Y, Jiang Z, Kema G, Li X, Liu F, Liu H, Liu Y, Lu Q, Luo Q, Mao Y, Msuya FE, Rebours C, Shen H, Stentiford GD, Yarish C, Wu H, Yang X, Zhang J, Zhou Y, Gachon CMM (2016) Safeguarding the future of the global seaweed aquaculture industry. United Nations University. https://www.sams.ac.uk/t4-media/sams/pdf/globalseaweed-policy-brief.pdf

[CR30] Craigie JS, Correa JA (1996) Etiology of infectious diseases in cultivated *Chondrus crispus* (Gigartinales, Rhodophyta). Hydrobiologia 326:97–104

[CR31] Croft MT, Lawrence AD, Raux-Deery E, Warren MJ, Smith AG (2005) Algae acquire vitamin B_12_ through a symbiotic relationship with bacteria. Nature 438:90–93 10.1038/nature04056 16267554

[CR32] Da Gama B, Plouguerné E, Pereira R (2014) The antifouling defence mechanisms of marine macroalgae. Adv Bot Res 71:413–440

[CR33] Dai J, Zhang Q, Bao Z (1993) Genetic breeding and seedling raising experiments with *Porphyra* protoplasts. Aquaculture 111:139–145

[CR34] Davis TA, Volesky B, Mucci A (2003) A review of the biochemistry of heavy metal biosorption by brown algae. Water Res 37:4311–4330 14511701 10.1016/S0043-1354(03)00293-8

[CR35] de Oliveira LS, Gregoracci GB, Silva GGZ, Salgado LT, Filho GA, Alves-Ferreira M, Pereira RC, Thompson FL (2012) Transcriptomic analysis of the red seaweed *Laurencia dendroidea* (Florideophyceae, Rhodophyta) and its microbiome. BMC Genomics 13:487 22985125 10.1186/1471-2164-13-487 PMC3534612

[CR36] de Oliveira LS, Tschoeke DA, Magalhães Lopes ACR, Sudatti DB, Meirelles PM, Thompson CC, Pereira RC, Thompson FL (2017) Molecular mechanisms for microbe recognition and defense by the red seaweed *Laurencia dendroidea*. mSphere 2:e00094-17 29242829 10.1128/mSphere.00094-17 PMC5717322

[CR37] DeWeese KJ, Osborne MG (2021) Understanding the metabolome and metagenome as extended phenotypes: the next frontier in macroalgae domestication and improvement. J World Aquac Soc 52:1009–1030 34732977 10.1111/jwas.12782 PMC8562568

[CR38] Dhiman S, Ulrich JF, Wienecke P, Wichard T, Arndt HD (2022) Stereoselective total synthesis of (−)-thallusin for bioactivity profiling. Angew Chem Int Ed 61:e202206746 10.1002/anie.202206746 PMC9804709 35900916

[CR39] Dimitroglou A, Merrifield DL, Carnevali O, Picchietti S, Avella M, Daniels C, Güroy D, Davies SJ (2011) Microbial manipulations to improve fish health and production – a Mediterranean perspective. Fish Shellfish Immunol 30:1–16 20801223 10.1016/j.fsi.2010.08.009

[CR40] Dittami SM, Duboscq-Bidot L, Perennou M, Gobet A, Corre E, Boyen C, Tonon T (2016) Host–microbe interactions as a driver of acclimation to salinity gradients in brown algal cultures. ISME J 10:51–63 26114888 10.1038/ismej.2015.104 PMC4681850

[CR41] Dobretsov SV, Qian PY (2002) Effect of bacteria associated with the green alga *Ulva reticulata* on marine micro- and macrofouling. Biofouling 18:217–228

[CR42] Duarte CM, Wu J, Xiao X, Bruhn A, Krause-Jensen D (2017) Can seaweed farming play a role in climate change mitigation and adaptation? Front Mar Sci 4:100

[CR43] Dworjanyn S, de Nys R, Steinberg P (2006) Chemically mediated antifouling in the red alga *Delisea pulchra*. Mar Ecol Prog Ser 318:153–163

[CR44] Egan S, Fernandes ND, Kumar V, Gardiner M, Thomas T (2014) Bacterial pathogens, virulence mechanism and host defence in marine macroalgae. Environ Microbiol 16:925–938 24112830 10.1111/1462-2920.12288

[CR45] Egan S, Gardiner M (2016) Microbial dysbiosis: rethinking disease in marine ecosystems. Front Microbiol 7:991 27446031 10.3389/fmicb.2016.00991 PMC4914501

[CR46] Egan S, Harder T, Burke C, Steinberg P, Kjelleberg S, Thomas T (2013) The seaweed holobiont: understanding seaweed–bacteria interactions. FEMS Microbiol Rev 37:462–476 23157386 10.1111/1574-6976.12011

[CR47] Eger AM, Aguirre JD, Altamirano M, Arafeh-Dalmau N, Arroyo NL et al (2024) The kelp forest challenge: a collaborative global movement to protect and restore 4 million hectares of kelp forests. J Appl Phycol 36:951–964

[CR48] El-Saadony MT, Alagawany M, Patra AK, Kar I, Tiwari R, Dawood MAO, Dhama K, Abdel-Latif HMR (2021) The functionality of probiotics in aquaculture: an overview. Fish Shellfish Immunol 117:36–52 34274422 10.1016/j.fsi.2021.07.007

[CR49] Ezura Y, Yamamoto H, Kimura T (1988) Isolation of a marine bacterium that produces red-spots on the culture bed of *Makonbu Laminaria japonica* cultivation. Nippon Suisan Gakkaishi 54:665–672

[CR50] Fan X, Ye T, Li Q, Bhatt P, Zhang L, Chen S (2020) Potential of a quorum quenching bacteria isolate *Ochrobactrum intermedium* D-2 against soft rot pathogen *Pectobacterium carotovorum* subsp. *carotovorum*. Front Microbiol 11:898 32457732 10.3389/fmicb.2020.00898 PMC7227377

[CR51] FAO (2018) The global status of seaweed production, trade and utilization. Food and Agriculture Organization of the United Nations, Rome

[CR52] FAO (2020) The state of world fisheries and aquaculture 2020. Sustainability in action. Food and Agriculture Organization of the United Nations, Rome

[CR53] FAO (2024) The state of world fisheries and aquaculture 2024: Blue transformation in action. Food and Agriculture Organization of the United Nations, Rome

[CR54] Fei X (2004) Solving the coastal eutrophication problem by large scale seaweed cultivation. Hydrobiologia 512:145–151

[CR55] Feng X, Xiao B, Jiang M, Li P, Wu Q, Dong Y, Wang J, Sui Z (2023) Identification of candidate genes related to two economic traits using GWAS in *Gracilariopsis lemaneiformis* (Rhodophyta). Algal Res 76:103309

[CR56] Fernandes ND, Case RJ, Longford SR, Seyedsayamdost MR, Steinberg P, Kjelleberg S, Thomas T (2011) Genomes and virulence factors of novel bacterial pathogens causing bleaching disease in the marine red alga *Delisea pulchra*. PLoS One 6:e27387 22162749 10.1371/journal.pone.0027387 PMC3230580

[CR57] Fernandes ND, Steinberg P, Rusch D, Kjelleberg S, Thomas T (2012) Community structure and functional gene profile of bacteria on healthy and diseased thalli of the red seaweed *Delisea pulchra*. PLoS One 7:e50854 23226544 10.1371/journal.pone.0050854 PMC3513314

[CR58] Fiorenza EA, Abu N, Feeney WE, Limbong SR, Freimark CB, Jompa J, Harvel CD, Lamb JB (2024) Seagrass ecosystems reduce disease risk and economic loss in marine farming production. Proc Natl Acad Sci USA 121:e2416012121 39680762 10.1073/pnas.2416012121 PMC11670088

[CR59] Foster KR, Schluter J, Coyte KZ, Rakoff-Nahoum S (2017) The evolution of the host microbiome as an ecosystem on a leash. Nature 548:43–51 28770836 10.1038/nature23292 PMC5749636

[CR60] Fouqueau L, Reynes L, Tempera F, Bajjouk T, Blanfuné A, Chevalier C, Laurans M, Mauger S, Sourisseau M, Assis J, Lévêque L, Valero M (2024) Seascape genetic study on *Laminaria digitata* underscores the critical role of sampling schemes. Mar Ecol Prog Ser 740:23–42

[CR61] Fu X, Wu X, Zhou X, Liu S, Shen Y, Wu F (2015) Companion cropping with potato onion enhances the disease resistance of tomato against *Verticillium dahliae*. Front Plant Sci 6:726 26442040 10.3389/fpls.2015.00726 PMC4566073

[CR62] Fujita J (1990) Diseases of cultivated *Porphyra* in Japan. In: Akatsuka I (ed) Introduction to applied phycology. SPB Academic, The Hague, pp 177–190

[CR63] Gachon CMM, Sime-Ngando T, Strittmatter M, Chambouvet A, Kim GH (2010) Algal diseases: spotlight on a black box. Trends Plant Sci 15:633–640 20833575 10.1016/j.tplants.2010.08.005

[CR64] Ganguly S, Dora KC, Sarkar S, Chowdhury S (2013) Supplementation of prebiotics in fish feed: a review. Rev Fish Biol Fish 23:195–199

[CR65] Gao G, Gao L, Jiang M, Jian A, He L (2021) The potential of seaweed cultivation to achieve carbon neutrality and mitigate deoxygenation and eutrophication. Environ Res Lett 17:014018

[CR66] Gardiner M, Bournazos AM, Maturana-Martinez C, Zhong L, Egan S (2017) Exoproteome analysis of the seaweed pathogen *Nautella italica* R11 reveals temperature-dependent regulation of RTX-like proteins. Front Microbiol 8:1203 28706511 10.3389/fmicb.2017.01203 PMC5489592

[CR67] Gardiner M, Fernandes ND, Nowakowski D, Raftery M, Kjelleberg S, Zhong L, Thomas T, Egan S (2015a) VarR controls colonization and virulence in the marine macroalgal pathogen *Nautella italica* R11. Front Microbiol 6:1130 26528274 10.3389/fmicb.2015.01130 PMC4602140

[CR68] Gardiner M, Thomas T, Egan S (2015b) A glutathione peroxidase (GpoA) plays a role in the pathogenicity of *Nautella italica* strain R11 towards the red alga *Delisea pulchra*. FEMS Microbiol Ecol 91:fiv021 25764469 10.1093/femsec/fiv021

[CR69] Ghaderiardakani F, Califano G, Mohr J, Abreu MH, Coates J, Wichard T (2019) Analysis of algal growth- and morphogenesis- promoting factors in an integrated multi-trophic aquaculture system for farming *Ulva* spp. Aquac Environ Interact 11:375–391

[CR70] Ghaderiardakani F, Quartino ML, Wichard T (2020) Microbiome-dependent adaptation of seaweeds under environmental stresses: a perspective. Front Mar Sci 7:575228

[CR71] Goecke F, Klemetsdal G, Ergon Å (2020) Cultivar development of kelps for commercial cultivation—past lessons and future prospects. Front Mar Sci 7:110

[CR72] González JE, Keshavan ND (2006) Messing with bacterial quorum sensing. Microbiol Mol Biol Rev 70:859–875 17158701 10.1128/MMBR.00002-06 PMC1698510

[CR73] Granato ET, Meiller-Legrand TA, Foster KR (2019) The evolution and ecology of bacterial warfare. Curr Biol 29:R521–R537 31163166 10.1016/j.cub.2019.04.024

[CR74] Gu S, Wei Z, Shao Z, Friman V-P, Cao K, Yang T, Kramer J, Wang X, Li M, Mei X, Xu Y, Shen Q, Kümmerli R, Jousset A (2020) Competition for iron drives phytopathogen control by natural rhizosphere microbiomes. Nat Microbiol 5:1002–1010 32393858 10.1038/s41564-020-0719-8 PMC7116525

[CR75] Hardegen J, Amend G, Wichard T (2023) Lifecycle-dependent toxicity and removal of micropollutants in algal cultures of the green seaweed *Ulva* (Chlorophyta). J Appl Phycol 35:2031–2048

[CR76] Harder T, Campbell AH, Egan S, Steinberg PD (2012) Chemical mediation of ternary interactions between marine holobionts and their environment as exemplified by the red alga *Delisea pulchra*. J Chem Ecol 38:442–450 22527059 10.1007/s10886-012-0119-5

[CR77] Harvell CD, Altizer S, Cattadori IM, Harrington L, Weil E (2009) Climate change and wildlife diseases: when does the host matter the most? Ecology 90:912–920 19449685 10.1890/08-0616.1

[CR78] Harvell CD, Kim K, Burkholder JM, Colwell RR, Epstein PR, Grimes DJ, Hofmann EE, Lipp EK, Osterhaus AD, Overstreet RM, Porter JW, Smith GW, Vasta GR (1999) Emerging marine diseases–climate links and anthropogenic factors. Science 285:1505–1510 10498537 10.1126/science.285.5433.1505

[CR79] Harvell CD, Mitchell CE, Ward JR, Altizer S, Dobson AP, Ostfeld RS, Samuel MD (2002) Climate warming and disease risks for terrestrial and marine biota. Science 296:2158–2162 12077394 10.1126/science.1063699

[CR80] Hasselström L, Visch W, Gröndahl F, Nylund GM, Pavia H (2018) The impact of seaweed cultivation on ecosystem services - a case study from the west coast of Sweden. Mar Pollut Bull 133:53–64 30041346 10.1016/j.marpolbul.2018.05.005

[CR81] Hedberg N, von Schreeb K, Charisiadou S, Jiddawi NS, Tedengren M, Nordlund LM (2018) Habitat preference for seaweed farming – a case study from Zanzibar, Tanzania. Ocean Coast Manag 154:186–195

[CR82] Heinrich S, Valentin K, Frickenhaus S, John U, Wiencke C (2012) Transcriptomic analysis of acclimation to temperature and light stress in *Saccharina latissima* (Phaeophyceae). PLoS One 7:e44342 22937172 10.1371/journal.pone.0044342 PMC3429442

[CR83] Hermans (2021) State of the seaweed industry 2022, Phyconomy. https://phyconomy.net/articles/state-of-the-seaweed-industry-2022/; accessed 30 September 2025

[CR84] Hmani I, Ghaderiardakani F, Ktari L, Bour ME, Wichard T (2024) High-temperature stress induces bacteria-specific adverse and reversible effects on *Ulva* (Chlorophyta) growth and its chemosphere in a reductionist model system. Bot Mar 67:131–138

[CR85] Hofmann LC, Brakel J, Bartsch I, Montecinos Arismendi G, Bermejo R, Parente MI, Creis E, De Clerck O, Jacquemin B, Knoop J, Lorenz M, Machado LP, Martins N, Orfanidis S, Probert I, Rad Menendez C, Ross M, Rautenberger R, Schiller J, Serrao EA, Steinhagen S, Sulpice R, Valero M, Wichard T (2025) A European biobanking strategy for safeguarding macroalgal genetic material to ensure food security, biosecurity and conservation of biodiversity. Eur J Phycol 60:197–220

[CR86] Hooks KB, O’Malley MA (2017) Dysbiosis and its discontents. mBio 8:e01492–17 10.1128/mBio.01492-17 PMC5635691 29018121

[CR87] Huang X, Han B (2014) Natural variations and genome-wide association studies in crop plants. Annu Rev Plant Biol 65:531–551 24274033 10.1146/annurev-arplant-050213-035715

[CR88] Huang X, Yang S, Gong J, Zhao Y, Feng Q, Gong H, Li W, Zhan Q, Cheng B, Xia J, Chen N, Hao Z, Liu K, Zhu C, Huang T, Zhao Q, Zhang L, Fan D, Zhou C, Lu Y, Weng Q, Wang Z-X, Li J, Han B (2015) Genomic analysis of hybrid rice varieties reveals numerous superior alleles that contribute to heterosis. Nat Commun 6:6258 25651972 10.1038/ncomms7258 PMC4327311

[CR89] Hudson J, Gardiner M, Deshpande N, Egan S (2018) Transcriptional response of *Nautella italica* R11 towards its macroalgal host uncovers new mechanisms of host–pathogen interaction. Mol Ecol 27:1820–1832 29215165 10.1111/mec.14448

[CR90] Hurd CL, Law CS, Bach LT, Britton D, Hovenden M, Paine ER, Raven JA, Tamsitt V, Boyd PW (2022) Forensic carbon accounting: assessing the role of seaweeds for carbon sequestration. J Phycol 58:347–363 35286717 10.1111/jpy.13249

[CR91] Hurtado AQ, Neish IC, Critchley AT (2019) Phyconomy: the extensive cultivation of seaweeds, their sustainability and economic value, with particular reference to important lessons to be learned and transferred from the practice of eucheumatoid farming. Phycologia 58:472–483

[CR92] Hwang EK, Yotsukura N, Pang SJ, Su L, Shan TF (2019) Seaweed breeding programs and progress in eastern Asian countries. Phycologia 58:484–495

[CR93] Irmawati Y, Sudjiro F (2017) Infection *Vibrio* sp. bacteria on *Kappaphycus* seaweed varieties brown and green. IOP Conf Ser: Earth Environ Sci 89 :012016

[CR94] Jiang P, Qin S, Tseng C (2002) Expression of hepatitis B surface antigen gene (HBsAg) in *Laminaria japonica* (Laminariales, Phaeophyta). Chin Sci Bull 47:1438–1440

[CR95] Johari SA, Kalbassi MR, Soltani M, Yu IJ (2016) Application of nanosilver-coated zeolite as water filter media for fungal disinfection of rainbow trout (*Oncorhynchus mykiss*) eggs. Aquacult Int 24:23–38

[CR96] Jueterbock A, Minne AJP, Cock JM, Coleman MA, Wernberg T, Scheschonk L, Rautenberger R, Zhang J, Hu Z-M (2021) Priming of marine macrophytes for enhanced restoration success and food security in future oceans. Front Mar Sci 8:658485

[CR97] Ke J, Wang B, Yoshikuni Y (2021) Microbiome engineering: synthetic biology of plant-associated microbiomes in sustainable agriculture. Trends Biotechnol 39:244–261 32800605 10.1016/j.tibtech.2020.07.008

[CR98] Kessler RW, Weiss A, Kuegler S, Hermes C, Wichard T (2018) Macroalgal–bacterial interactions: role of dimethylsulfoniopropionate in microbial gardening by *Ulva* (Chlorophyta). Mol Ecol 27:1808–1819 29290092 10.1111/mec.14472

[CR99] Kim GH, Klochkova TA, Lee DJ, Im SH (2016) Chloroplast virus causes green-spot disease in cultivated *Pyropia* of Korea. Algal Res 17:293–299

[CR100] Kim GH, Moon K-H, Kim J-Y, Shim J, Klotchkova TA (2014) A revaluation of algal diseases in Korean *Pyropia* (*Porphyra*) sea farms and their economic impact. Algae 29:249–265

[CR101] King NG, Moore PJ, Thorpe JM, Smale DA (2023) Consistency and variation in the kelp microbiota: patterns of bacterial community structure across spatial scales. Microb Ecol 85:1265–1275 35589992 10.1007/s00248-022-02038-0

[CR102] Koch R (1891) Über Bakteriologische Forschung. Dtsch Med Wochenschr 16:757–758

[CR103] Kumar V, Zozaya-Valdes E, Kjelleberg S, Thomas T, Egan S (2016) Multiple opportunistic pathogens can cause a bleaching disease in the red seaweed *Delisea pulchra*. Environ Microbiol 18:3962–3975 27337296 10.1111/1462-2920.13403

[CR104] Küpper F, Kloareg B, Guern J, Potin P (2001) Oligoguluronates elicit an oxidative burst in the brown algal kelp *Laminaria digitata*. Plant Physiol 125:278–291 11154336 10.1104/pp.125.1.278 PMC61009

[CR105] Küpper FC, Gaquerel E, Cosse A, Adas F, Peters AF, Müller DG, Kloareg B, Salaün J-P, Potin P (2009) Free fatty acids and methyl jasmonate trigger defense reactions in *Laminaria digitata*. Plant Cell Physiol 50:789–800 19213737 10.1093/pcp/pcp023

[CR106] Kusuda R, Kawai K, Salati F, Kawamura Y, Yamashita Y (1992) Characteristics of *Flavobacterium* sp. causing “Suminori” disease in cultivated *Porphyra*. Aquac Sci 40:457–461

[CR107] Lachnit T, Blümel M, Imhoff J, Wahl M (2009) Specific epibacterial communities on macroalgae: phylogeny matters more than habitat. Aquat Biol 5:181–186

[CR108] Lang T, Cummins SF, Paul NA, Campbell AH (2024) Molecular responses of seaweeds to biotic interactions: a systematic review. J Phycol 60:1036–1057 39298370 10.1111/jpy.13504

[CR109] Largo DB (2002) Recent developments in seaweed diseases. In: Proceedings of the National Seaweed Planning Workshop held on August 2–3, 2001, SEADFEC Aquaculture Department, Tigbauan, Iloilo, pp 35–42

[CR110] Largo DB, Fukami K, Nishijima T (1995a) Occasional pathogenic bacteria promoting ice-ice disease in the carrageenan-producing red algae *Kappaphycus alvarezii* and *Eucheuma denticulatum* (Solieriaceae, Gigartinales, Rhodophyta). J Appl Phycol 7:545–554

[CR111] Largo DB, Fukami K, Nishijima T (1999) Time-dependent attachment mechanism of bacterial pathogen during ice-ice infection in *Kappaphycus alvarezii* (Gigartinales, Rhodophyta). J Appl Phycol 11:129–136

[CR112] Largo DB, Fukami K, Nishijima T, Ohno M (1995b) Laboratory-induced development of the ice-ice disease of the farmed red algae *Kappaphycus alvarezii* and *Eucheuma denticulatum* (Solieriaceae, Gigartinales, Rhodophyta). J Appl Phycol 7:539–543

[CR113] Lau S-E, Teo WFA, Teoh EY, Tan BC (2022) Microbiome engineering and plant biostimulants for sustainable crop improvement and mitigation of biotic and abiotic stresses. Discov Food 2:9 10.3390/plants11192625 PMC9573444 36235491

[CR114] Lavilla-Pitogo CR (1992) Agar-digesting bacteria associated with ‘rotten thallus syndrome’ of *Gracilaria* sp. Aquaculture 102:1–7

[CR115] Li J, Majzoub ME, Marzinelli EM, Dai Z, Thomas T, Egan S (2022a) Bacterial controlled mitigation of dysbiosis in a seaweed disease. ISME J 16:378–387 34341505 10.1038/s41396-021-01070-1 PMC8776837

[CR116] Li J, Saha M, Majzoub ME, Yang T, Chu H, Thomas T, Weinberger F, Egan S (2024) Non-selective microbiota reduction after the elicitation of a seaweed’s immune response. Environ Microbiol Rep 16:e13268 38761002 10.1111/1758-2229.13268 PMC11101764

[CR117] Li J, Weinberger F, de Nys R, Thomas T, Egan S (2023) A pathway to improve seaweed aquaculture through microbiota manipulation. Trends Biotechnol 41:545–556 36089422 10.1016/j.tibtech.2022.08.003

[CR118] Li J, Weinberger F, Saha M, Majzoub ME, Egan S (2022b) Cross-host protection of marine bacteria against macroalgal disease. Microb Ecol 84:1288–1293 34731271 10.1007/s00248-021-01909-2

[CR119] Li X, Cong Y, Yang G, Shi Y, Qu S, Li Z, Wang G, Zhang Z, Luo S, Dai H, Xie J, Jiang G, Liu J, Wang T (2007) Trait evaluation and trial cultivation of Dongfang No. 2, the hybrid of a male gametophyte clone of *Laminaria longissima* (Laminariales, Phaeophyta) and a female one of *L. japonica*. J Appl Phycol 19:139–151 19396352 10.1007/s10811-006-9120-0 PMC2668635

[CR120] Ling F, Egan S, Zhuang Y, Chang L, Xiao L, Yang Q, Wang G (2022) Epimicrobiome shifts with bleaching disease progression in the brown seaweed *Saccharina japonica*. Front Mar Sci 9:865224

[CR121] Littler MM, Littler DS (1994) A pathogen of reef-building coralline algae discovered in the South Pacific. Coral Reefs 13:202–202

[CR122] Littler MM, Littler DS (1995) Impact of CLOD pathogen on Pacific coral reefs. Science 267:1356–1359 17812612 10.1126/science.267.5202.1356

[CR123] Liu C, Wang L, Wang M, Tang X (2002) Difference analysis of infection activity of alginic acid decomposing bacteria infecting *Laminaria japonica*. Mar Sci 6:44–47

[CR124] Liu Q, Shi H, Yang R, Lin J, Chen J, Chen H (2025) *Phaeobacter italicus* JN-W1: a pathogenic bacterium causing bleaching disease in *Porphyra* sensu lato (Bangiales, Rhodophyta). Aquaculture 595:741641

[CR125] Liu X, Chen Y, Zhong M, Chen W, Lin Q, Du H (2019) Isolation and pathogenicity identification of bacterial pathogens in bleached disease and their physiological effects on the red macroalga *Gracilaria lemaneiformis*. Aquat Bot 153:1–7

[CR126] Lomartire S, Marques JC, Gonçalves AMM (2021) An overview to the health benefits of seaweeds consumption. Mar Drugs 19:341 34203804 10.3390/md19060341 PMC8232781

[CR127] Lüning K (1991) Seaweeds: Their environment, biogeography, and ecophysiology. John Wiley & Sons, NY

[CR128] Ma M, Zhuang Y, Chang L, Xiao L, Lin Q, Qiu Q, Chen D, Egan S, Wang G (2023) Naturally occurring beneficial bacteria *Vibrio alginolyticus* X-2 protects seaweed from bleaching disease. Mbio 14:e00065-23 37310733 10.1128/mbio.00065-23 PMC10470739

[CR129] Makame MO, Hamad AR, Said MS, Mushi A, Sharif K (2021) Moving seaweed farms from shallow to deep seawater to cope with warming and diseases in Zanzibar. Current socio-economic and cultural barriers. J Sustain Dev 14:29

[CR130] Mancuso FP, Morrissey KL, De Clerck O, Airoldi L (2023) Warming and nutrient enrichment can trigger seaweed loss by dysregulation of the microbiome structure and predicted function. Sci Total Environ 879:162919 36958561 10.1016/j.scitotenv.2023.162919

[CR131] Manefield M, Welch M, Givskov M, Salmond G, Kjelleberg S (2011) Halogenated furanones from the red alga, *Delisea pulchra*, inhibit carbapenem antibiotic synthesis and exoenzyme virulence factor production in the phytopathogen *Erwinia carotovora*. FEMS Microbiol Lett 2015:131–138 10.1111/j.1574-6968.2001.tb10936.x 11728727

[CR132] Mao X, Augyte S, Huang M, Hare MP, Bailey D, Umanzor S, Marty-Rivera M, Robbins KR, Yarish C, Lindell S, Jannink J-L (2020) Population genetics of sugar kelp throughout the northeastern United States using genome-wide markers. Front Mar Sci 7:694

[CR133] Martins N, Barreto L, Bartsch I, Bernard J, Serrão E, Pearson G (2022) Daylength influences reproductive success and sporophyte growth in the Arctic kelp species *Alaria esculenta*. Mar Ecol Prog Ser 683:37–52

[CR134] Matsuo Y, Imagawa H, Nishizawa M, Shizuri Y (2005) Isolation of an algal morphogenesis inducer from a marine bacterium. Science 307:1598–1598 15761147 10.1126/science.1105486

[CR135] Matsuo Y, Suzuki M, Kasai H, Shizuri Y, Harayama S (2003) Isolation and phylogenetic characterization of bacteria capable of inducing differentiation in the green alga *Monostroma oxyspermum*. Environ Microbiol 5:25–35 12542710 10.1046/j.1462-2920.2003.00382.x

[CR136] Maximilien R, de Nys R, Holmström C, Gram L, Givskov M, Crass K, Kjelleberg S, Steinberg P (1998) Chemical mediation of bacterial surface colonisation by secondary metabolites from the red alga *Delisea pulchra*. Aquat Microb Ecol 15:233–246

[CR137] Mine T, Tanaka S, Kawamura Y, Kobayashi G, Kanda K (2009) Diversity of incidence factors in Suminori disease during Laver cultivation. Aquac Sci 57:601–608

[CR138] Mine T, Tanaka S, Kawamura Y, Kobayashi G, Kanda K (2010) Isolation and application of bacteriophages to Suminori disease control. Aquac Sci 58:211–217

[CR139] Mudlaff CM, Weinberger F, Düsedau L, Ghotbi M, Künzel S, Bonthond G (2025) Seasonal cycles in a seaweed holobiont: a multiyear time series reveals repetitive microbial shifts and core taxa. Environ Microbiol 27:e70062 40015318 10.1111/1462-2920.70062 PMC11867712

[CR140] Murray SC, Rooney WL, Mitchell SE, Sharma A, Klein PE, Mullet JE, Kresovich S (2008) Genetic improvement of Sorghum as a biofuel feedstock: II. QTL for stem and leaf structural carbohydrates. Crop Sci 48:2180–2193

[CR141] Nayar S, Bott K (2014) Current status of global cultivated seaweed production and markets. World Aquac 45:32–37

[CR142] Nelson DR, Mystikou A, Jaiswal A, Rad-Menendez C, Preston MJ, De Boever F, El Assal DC, Daakour S, Lomas MW, Twizere J-C, Green DH, Ratcliff WC, Salehi-Ashtiani K (2024) Macroalgal deep genomics illuminate multiple paths to aquatic, photosynthetic multicellularity. Mol Plant 17:747–771 38614077 10.1016/j.molp.2024.03.011

[CR143] Nguyen D, Ovadia O, Guttman L (2023) Temporal force governs the microbial assembly associated with *Ulva fasciata* (Chlorophyta) from an integrated multi-trophic aquaculture system. Front Microbiol 14:1223204 37869666 10.3389/fmicb.2023.1223204 PMC10585273

[CR144] Pang T, Liu J, Liu Q, Li H, Li J (2015) Observations on pests and diseases affecting a eucheumatoid farm in China. J Appl Phycol 27:1975–1984

[CR145] Park SR, Cho S, Kim M, Lim W, Ryu S, An C, Hong S, Lee Y, Yun H (2001) Characteristics of a marine agarolytic *Pseudomonas* sp. from *Porphyra dentata* (Bangiales, Rhodophyta) and some properties of its extracellular agarase. Kor J Life Sci 11:291–297

[CR146] Paasch BC, He SY (2021) Toward understanding microbiota homeostasis in the plant kingdom. PLoS Pathog 17:e2009472 10.1371/journal.ppat.1009472 PMC8061798 33886694

[CR147] Patwary ZP, Paul NA, Nishitsuji K, Campbell AH, Shoguchi E, Zhao M, Cummins SF (2021) Application of omics research in seaweeds with a focus on red seaweeds. Brief Funct Genomics 20:148–161 33907795 10.1093/bfgp/elab023

[CR148] Pearman WS, Duffy GA, Smith RO, Currie KI, Gemmell NJ, Morales SE, Fraser CI (2024) Host dispersal relaxes selective pressures in rafting microbiomes and triggers successional changes. Nat Commun 15:10759 39737966 10.1038/s41467-024-54954-z PMC11685921

[CR149] Peixoto RS, Voolstra CR, Sweet M, Duarte CM, Carvalho S, Villela H, Lunshof JE, Gram L, Woodhams DC, Walter J, Roik A, Hentschel U, Thurber RV, Daisley B, Ushijima B, Daffonchio D, Costa R, Keller-Costa T, Bowman JS, Rosado AS, Reid G, Mason CE, Walke JB, Thomas T, Berg G (2022) Harnessing the microbiome to prevent global biodiversity loss. Nat Microbiol 7:1726–1735 35864220 10.1038/s41564-022-01173-1

[CR150] Peng Y, Li W (2013) A bacterial pathogen infecting gametophytes of *Saccharina japonica* (Laminariales, Phaeophyceae). Chin J Ocean Limnol 31:366–373

[CR151] Pessarrodona A, Howard J, Pidgeon E, Wernberg T, Filbee-Dexter K (2024) Carbon removal and climate change mitigation by seaweed farming: a state of knowledge review. Sci Total Environ 918:170525 38309363 10.1016/j.scitotenv.2024.170525

[CR152] Provasoli L, Pintner IJ (1980) Bacteria induced polymorphism in an axenic laboratory strain of *Ulva lactuca* (Chlorophyceae). J Phycol 16:196–201

[CR153] Qui-Minet AN, Connan S, Stiger-Pouvreau V (2025) To die or not to die: how seaweed holobionts chemistry influences lifespan and stress resilience. Front Mar Sci 12:1635698

[CR154] Quiza L, St-Arnaud M, Yergeau E (2015) Harnessing phytomicrobiome signaling for rhizosphere microbiome engineering. Front Plant Sci 6:507 26236319 10.3389/fpls.2015.00507 PMC4500914

[CR155] Robinson N, Winberg P, Kirkendale L (2013) Genetic improvement of macroalgae: status to date and needs for the future. J Appl Phycol 25:703–716

[CR156] Rogers AB (2012) Gastric *Helicobacter* spp. in animal models: pathogenesis and modulation by extragastric coinfections. Methods Mol Biol 921:175–188 23015504 10.1007/978-1-62703-005-2_21

[CR157] Roque BM, Venegas M, Kinley RD, de Nys R, Duarte TL, Kebreab E (2021) Red seaweed *(Asparagopsis taxiformis*) supplementation reduces enteric methane by over 80 percent in beef steers. PLoS One 16:e0247820 33730064 10.1371/journal.pone.0247820 PMC7968649

[CR158] Saha M, Rempt M, Grosser K, Pohnert G, Weinberger F (2011) Surface-associated fucoxanthin mediates settlement of bacterial epiphytes on the rockweed *Fucus vesiculosus*. Biofouling 27:423–433 21547758 10.1080/08927014.2011.580841

[CR159] Saha M, Weinberger F (2019) Microbial “gardening” by a seaweed holobiont: surface metabolites attract protective and deter pathogenic epibacterial settlement. J Ecol 107:2255–2265

[CR160] Saha M, Dittami SM, Chan CX, Raina JB, Stock W, Ghaderiardakani F, Valathuparambil Baby John AM, Corr S, Schleyer G, Todd J, Cardini U, Bengtsson MM, Prado S, Skillings D, Sonnenschein EC, Engelen AH, Wang G, Wichard T, Brodie J, Leblanc C, Egan S (2024) Progress and future directions for seaweed holobiont research. New Phytol 24:364–376 10.1111/nph.20018 39137959

[CR161] Sawabe T, Makino H, Tatsumi M, Nakano K, Tajima K, Iqbal M, Yumoto I, Ezura Y, Christen R (1998) *Pseudoalteromonas bacteriolytica* sp. nov., a marine bacterium that is the causative agent of red spot disease of *Laminaria japonica*. Int J Syst Evol Microbiol 48:769–774 10.1099/00207713-48-3-769 9734030

[CR162] Schroeder DC, Jaffer MA, Coyne VE (2003) Investigation of the role of a *β*(1–4) agarase produced by *Pseudoalteromonas gracilis* B9 in eliciting disease symptoms in the red alga *Gracilaria gracilis*. Microbiology 149:2919–2929 10.1099/mic.0.26513-0 14523124

[CR163] Shen Y, Motomura T, Ichihara K, Matsuda Y, Yoshimura K, Kosugi C, Nagasato C (2023) Application of CRISPR-Cas9 genome editing by microinjection of gametophytes of *Saccharina japonica* (Laminariales, Phaeophyceae). J Appl Phycol 35:1431–1441

[CR164] Simon J-C, Marchesi JR, Mougel C, Selosse M-A (2019) Host-microbiota interactions: from holobiont theory to analysis. Microbiome 7:5 30635058 10.1186/s40168-019-0619-4 PMC6330386

[CR165] Spagnuolo D, Genovese G (2024) Macroalgal diseases: exploring biology, pathogenesis, and management strategies. Phycology 4:450–464

[CR166] Strittmatter M, Murúa P, Arce P, Perrineau M-M, Gachon C (2022) My seaweed looks weird: a community web portal to accelerate pathogen discovery in seaweeds. Appl Phycol 3:300–305

[CR167] Sugumaran R, Padam BS, Yong WTL, Saallah S, Ahmed K, Yusof NA (2022) A retrospective review of global commercial seaweed production—current challenges, biosecurity and mitigation measures and prospects. Int J Environ Res Public Health 19:7087 35742332 10.3390/ijerph19127087 PMC9222978

[CR168] Sun X, He Y, Xu N, Xia Y, Liu Z (2012) Isolation and identification of two strains of pathogenic bacteria and their effects on the volatile metabolites of *Gracilariopsis lemaneiformis* (Rhodophyta). J Appl Phycol 24:277–284

[CR169] Sunairi M, Tsuchiya H, Tsuchiya T, Omura Y, Koyanagi Y, Ozawa M, Iwabuchi N, Murooka H, Nakajima M (1995) Isolation of a bacterium that causes Anaaki disease of the red algae *Porphyra yezoensis*. J Appl Bacteriol 79:225–229

[CR170] Syafitri E, Prayitno SB, Ma’ruf WF, Radjasa OK (2017) Genetic diversity of the causative agent of ice-ice disease of the seaweed *Kappaphycus alvarezii* from Karimunjawa island, Indonesia. IOP Conf Ser: Earth Environ Sci 55

[CR171] Tang L, Qiu L, Liu C, Du G, Mo Z, Tang X, Mao Y (2019) Transcriptomic insights into innate immunity responding to red rot disease in red alga *Pyropia yezoensis*. Int J Mol Sci 20:5970 31783543 10.3390/ijms20235970 PMC6928737

[CR172] Tay WH, Chong KKL, Kline (2016) Polymicrobial–host interactions during infection. J Mol Biol 428:3355–3371 10.1016/j.jmb.2016.05.006 27170548

[CR173] Thien VY, Yong WTL, Anton A, Chin GJWL (2020) A multiplex PCR method for rapid identification of commercially important seaweeds *Kappaphycus alvarezii, Kappaphycus striatus* and *Eucheuma denticulatum* (Rhodophyta, Solieriaceae). Reg Stud Mar Sci 40:101499

[CR174] Thomas J-BE, Ramos FS, Gröndahl F (2019) Identifying suitable sites for macroalgae cultivation on the Swedish West Coast. Coast Manag 47:88–106

[CR175] Toth GB, Pavia H (2000) Water-borne cues induce chemical defense in a marine alga (*Ascophyllum nodosum*). Proc Natl Acad Sci USA 97:14418–14420 11106371 10.1073/pnas.250226997 PMC18933

[CR176] Tracy AM, Pielmeier ML, Yoshioka RM, Heron SF, Harvell CD (2019) Increases and decreases in marine disease reports in an era of global change. Proc R Soc B 286:20191718 31594507 10.1098/rspb.2019.1718 PMC6790777

[CR177] Troell M, Joyce A, Chopin T, Neori A, Buschmann AH, Fang J-G (2009) Ecological engineering in aquaculture — potential for integrated multi-trophic aquaculture (IMTA) in marine offshore systems. Aquaculture 297:1–9

[CR178] Troell M, Henriksson PJG, Buschmann AH, Chopin T, Quahe S (2022) Farming the ocean – seaweeds as a quick fix? Rev Fish Sci Aquac 31:285–295

[CR179] Trujillo E, Monreal-Escalante E, Ramos-Vega A, Angulo C (2024) Macroalgae: marine players in vaccinology. Algal Res 78:103392

[CR180] Turnbaugh PJ, Hamady M, Yatsunenko T, Cantarel BL, Duncan A, Ley RE, Sogin ML, Jones WJ, Roe BA, Affourtit JP, Egholm M, Henrissat B, Heath AC, Knight R, Gordon JI (2009) A core gut microbiome in obese and lean twins. Nature 457:480–484 19043404 10.1038/nature07540 PMC2677729

[CR181] Ulrich JF, Gräfe MS, Dhiman S, Wienecke P, Arndt HD, Wichard T (2022) Thallusin quantification in marine bacteria and algae cultures. Mar Drugs 20:690 36355014 10.3390/md20110690 PMC9696546

[CR182] Uyenco FR, Saniel LS, Jacinto GS (1980) The “Ice-Ice” problem in seaweed farming. In: Levrig T (ed) International Seaweed Symposium: Proceedings. Göteborg, Sweden. Walter de Gruyter, Berlin, pp 625–630

[CR183] Vairappan CS, Suzuki M, Motomura T, Ichimura T (2001) Pathogenic bacteria associated with lesions and thallus bleaching symptoms in the Japanese kelp *Laminaria religiosa* Miyabe (Laminariales, Phaeophyceae). Hydrobiologia 445:183–191

[CR184] van der Loos LM, D’hondt S, Engelen AH, Pavia H, Toth GB, Willems A, Weinberger F, De Clerck O, Steinhagen S (2022) Salinity and host drive *Ulva*-associated bacterial communities across the Atlantic-Baltic Sea gradient. Mol Ecol 32:6260–6277 35395701 10.1111/mec.16462

[CR185] Vayssier-Taussat M, Albina E, Citti C, Cosson JF, Jacques M-A, Lebrun M-H, Le Loir Y, Ogliastro M, Petit M-A, Roumagnac P, Candresse T (2014) Shifting the paradigm from pathogens to pathobiome: new concepts in the light of meta-omics. Front Cell Infect Microbiol 4:29 24634890 10.3389/fcimb.2014.00029 PMC3942874

[CR186] Veenhof RJ, Burrows MT, Hughes AD, Michalek K, Ross ME, Thomson AI, Fedenko J, Stanley MS (2024) Sustainable seaweed aquaculture and climate change in the North Atlantic: challenges and opportunities. Front Mar Sci 11:1483330

[CR187] Voolstra CR, Raina J-B, Dörr M, Cárdenas A, Pogoreutz C, Silveira CB, Mohamed AR, Bourne DG, Luo H, Amin SA, Peixoto RS (2024) The coral microbiome in sickness, in health and in a changing world. Nat Rev Microbiol 22:460–475 38438489 10.1038/s41579-024-01015-3

[CR188] Vos M (2023) Accessory microbiomes. Microbiology 169:001332 37167086 10.1099/mic.0.001332 PMC10268833

[CR189] Wade R, Augyte S, Harden M, Nuzhdin SV, Yarish C, Alberto F (2020) Macroalgal germplasm banking for conservation, food security, and industry. PLoS Biol 18:e3000641 32058997 10.1371/journal.pbio.3000641 PMC7046291

[CR190] Wahl M (1989) Marine epibiosis. I. Fouling and antifouling: some basic aspects. Mar Ecol Prog Ser 58:175–189

[CR191] Wahl M, Goecke F, Labes A, Dobretsov S, Weinberger F (2012) The second skin: ecological role of epibiotic biofilms on marine organisms. Front Microbiol 3:292 22936927 10.3389/fmicb.2012.00292 PMC3425911

[CR192] Wang G, Lu B, Shuai L, Li D, Zhang R (2014) Microbial diseases of nursery and field-cultivated *Saccharina japonica* (Phaeophyta) in China. Algol Stud 145/146:39–51

[CR193] Wang G, Shuai L, Li Y, Lin W, Zhao X, Duan D (2008) Phylogenetic analysis of epiphytic marine bacteria on hole-rotten diseased sporophytes of *Laminaria japonica*. J Appl Phycol 20:403–409

[CR194] Wang X, Yao J, Zhang J, Duan D (2020) Status of genetic studies and breeding of *Saccharina japonica* in China. J Ocean Limnol 38:1064–1079

[CR195] Wang Y, Tang X, Yang Z, Yu Z (2006) Effect of alginic acid decomposing bacterium on the growth of *Laminaria japonica* (Phaeophyceae). J Environ Sci 18:543–551 17294654

[CR196] Ward GM, Faisan JJP, Cottier-Cook EJ, Gachon C, Hurtado AQ, Lim PE, Matoju I, Msuya FE, Bass D, Brodie J (2020) A review of reported seaweed diseases and pests in aquaculture in Asia. J World Aquac Soc 51:815–828

[CR197] Ward GM, Kambey CSB, Faisan JJP, Tan P-L, Daumich CC, Matoju I, Stentiford GD, Bass D, Lim P-E, Brodie J, Poong S-W (2022) Ice-ice disease: an environmentally and microbiologically driven syndrome in tropical seaweed aquaculture. Rev Aquac 14:414–439

[CR198] Weinberger F (1999) Epiphyte-host interactions: *Gracilaria conferta* (Rhodophyta) and associated bacteria. Doctoral dissertation, Christian-Albrechts Universitat

[CR199] Weinberger F (2007) Pathogen-induced defense and innate immunity in macroalgae. Biol Bull 213:290–302 18083968 10.2307/25066646

[CR200] Weinberger F, Friedlander M (2008) Response of *Gracilaria conferta* (Rhodophyta) to oligoagars results in defense against agar-degrading epiphytes. J Phycol 36:1079–1086

[CR201] Weinberger F, Friedlander M, Gunkel W (1994) A bacterial facultative parasite of *Gracilaria conferta*. Dis Aquat Org 18:135–141

[CR202] Weinberger F, Hoppe H-G, Friedlander M (1997) Bacterial induction and inhibition of a fast necrotic response in *Gracilaria conferta* (Rhodophyta). J Appl Phycol 9:277–285

[CR203] Wen T, Ding Z, Thomashow LS, Hale L, Yang S, Xie P, Liu X, Wang H, Shen Q, Yuan J (2023) Deciphering the mechanism of fungal pathogen-induced disease-suppressive soil. New Phytol 238:2634–2650 36932631 10.1111/nph.18886

[CR204] Weng P, Yang H, Mo Z, Zhang W, Yan Y, Rong X, Li J (2024) Application and evaluation of probiotics against red rot disease in *Pyropia*. Aquaculture 578:740050

[CR205] Wiese J, Thiel V, Nagel K, Staufenberger T, Imhoff JF (2009) Diversity of antibiotic-active bacteria associated with the brown alga *Laminaria saccharina* from the Baltic Sea. Mar Biotechnol 11:287–300 10.1007/s10126-008-9143-4 18855068

[CR206] Wilde J, Slack E, Foster KR (2024) Host control of the microbiome: mechanisms, evolution, and disease. Science 385:eadi3338 39024451 10.1126/science.adi3338

[CR207] Work TM, Aeby GS (2006) Systematically describing gross lesions in corals. Dis Aquat Org 70:155–160 10.3354/dao070155 16875402

[CR208] World Bank (2023) Global seaweed: New and emerging markers report 2023. World Bank, Washington, DC

[CR209] Wu C (1990) Cultivation of temperate seaweeds in the Asia Pacific region. Institute of Oceanology, Qingdao, China

[CR210] Wu L, Xiao W, Chen G, Song D, Khaskheli MA, Li P, Zhang S, Feng G (2018) Identification of *Pseudomonas mosselii* BS011 gene clusters required for suppression of Rice Blast Fungus *Magnaporthe oryzae*. J Biotechnol 282:1–9 29704539 10.1016/j.jbiotec.2018.04.016

[CR211] Xu M, Yang R, Liu Q, He Y, Chen H (2020) Yellow spot disease in *Pyropia* species infected by *Vibrio mediterranei* 117–T6. J Fish China 44:661–671

[CR212] Yamanoi H, Takami J (2008) A *Pseudomonas*-like bacterium isolated from diseased conchocelis of *Porphyra*. Nippon Suisan Gakkaishi 74:1024–1029

[CR213] Yang R, Liu Q, He Y, Tao Z, Xu M, Luo Q, Chen J, Chen H (2020) Isolation and identification of *Vibrio mediterranei* 117–T6 as a pathogen associated with yellow spot disease of *Pyropia* (Bangiales, Rhodophyta). Aquaculture 526:735372

[CR214] Yulianto H, Damai AA, Delis PC, Elisdiana Y (2017) Spatial analysis to evaluate the suitability of seaweed farming site in Lampung Bay, Indonesia. Turk J Fish Aquat Sci 17:1253–1261

[CR215] Yumoto I, Ezura Y, Kimura T (1989a) Distribution of the *Alteromonas* sp., the causative agent of red-spots on the culture bed of makonbu *Laminaria japonica*, in the coastal area of Funka Bay. Nippon Suisan Gakkaishi 55:453–462

[CR216] Yumoto I, Yamaguchi K, Yamada K, Ezura Y, Kimura T (1989b) Relationship between bacterial flora and occurrence of the *Alteromonas* sp., the causative agent of red-spots on the culture bed of makonbu *Laminaria japonica*, in the coastal area of Funka Bay. Nippon Suisan Gakkaishi 55:1907–1914

[CR217] Zainuddin EN, Anshary H, Huyyirnah H, Hiola R, Baxa DV (2019) Antibacterial activity of *Caulerpa racemosa* against pathogenic bacteria promoting “ice-ice” disease in the red alga *Gracilaria verrucosa*. J Appl Phycol 31:3201–3212

[CR218] Zhang X, Chen Y, Saha M, Zhuang Y, Chang L, Xiao L, Wang G (2022) *Pseudoalteromonas piscicida* X-8 causes bleaching disease in farmed *Saccharina japonica*. Aquaculture 546:737354 10.3390/biology12010047 PMC9855529 36671739

[CR219] Zhang Y, Jiang P, Gao J, Liao J, Sun S, Shen Z, Qin S (2008) Recombinant expression of rt-PA gene (encoding reteplase) in gametophytes of the seaweed *Laminaria japonica* (Laminariales, Phaeophyta). Sci China C Life Sci 51:1116–1120 19093086 10.1007/s11427-008-0143-4

[CR220] Zhang Y, Nair S, Zhang Z, Zhao J, Zhao H, Lu L, Chang L, Jiao N (2024) Adverse environmental perturbations may threaten kelp farming sustainability by exacerbating *Enterobacterales* disease. Environ Sci Technol 58:5796–5810 38507562 10.1021/acs.est.3c09921

[CR221] Zheng L, Han X, Chen H, Lin W, Yan X (2005) Marine bacteria associated with marine macroorganism: the potential antimicrobial resources. Ann Microbiol 55:119–124

[CR222] Zhuang Y, Liu T, Lin Q, Bai Y, Ma M, Wang M, Liu Q, Egan S, Wang G (2024) Transcriptome analysis reveals the rapid defense responses in a disease-resistant brown seaweed against a bacterial pathogen. Aquaculture 590:741024

[CR223] Zipfel C, Felix G (2005) Plants and animals: a different taste for microbes? Curr Opin Plant Biol 8:353–360 15922649 10.1016/j.pbi.2005.05.004

[CR224] Zozaya-Valdes E, Egan S, Thomas T (2015) A comprehensive analysis of the microbial communities of healthy and diseased marine macroalgae and the detection of known and potential bacterial pathogens. Front Microbiol 6:146 25759688 10.3389/fmicb.2015.00146 PMC4338804

